# Microbial species pool-mediated diazotrophic community assembly in crop microbiomes during plant development

**DOI:** 10.1128/msystems.01055-23

**Published:** 2024-03-19

**Authors:** Chao Xiong, Brajesh K. Singh, Yong-Guan Zhu, Hang-Wei Hu, Pei-Pei Li, Yan-Lai Han, Li-Li Han, Qin-Bing Zhang, Jun-Tao Wang, Si-Yi Liu, Chuan-Fa Wu, An-Hui Ge, Li-Mei Zhang, Ji-Zheng He

**Affiliations:** 1 State Key Laboratory of Urban and Regional Ecology, Research Center for Eco-Environmental Sciences, Chinese Academy of Sciences, Beijing, China; 2 University of Chinese Academy of Sciences, Beijing, China; 3 Hawkesbury Institute for the Environment, Western Sydney University, Penrith, New South Wales, Australia; 4 Global Centre for Land-Based Innovation, Western Sydney University, Penrith, New South Wales, Australia; 5 Faculty of Veterinary and Agricultural Sciences, The University of Melbourne, Parkville, Victoria, Australia; 6 College of Resource and Environmental Sciences, Henan Agricultural University, Zhengzhou, China; 7 Soil and Fertilizer Station of Qilin District, Qujing, Yunnan Province, China; Iowa State University, Ames, Iowa, USA

**Keywords:** plant microbiome, diazotrophs, cereal crops, microbial networks, soil–plant continuum, phyllosphere, metagenome-assembled genomes (MAGs), N-cycling pathways

## Abstract

Plant-associated diazotrophs strongly relate to plant nitrogen (N) supply and growth. However, our knowledge of diazotrophic community assembly and microbial N metabolism in plant microbiomes is largely limited. Here we examined the assembly and temporal dynamics of diazotrophic communities across multiple compartments (soils, epiphytic and endophytic niches of root and leaf, and grain) of three cereal crops (maize, wheat, and barley) and identified the potential N-cycling pathways in phylloplane microbiomes. Our results demonstrated that the microbial species pool, influenced by site-specific environmental factors (e.g., edaphic factors), had a stronger effect than host selection (i.e., plant species and developmental stage) in shaping diazotrophic communities across the soil–plant continuum. Crop diazotrophic communities were dominated by a few taxa (~0.7% of diazotrophic phylotypes) which were mainly affiliated with *Methylobacterium*, *Azospirillum*, *Bradyrhizobium*, and *Rhizobium*. Furthermore, eight dominant taxa belonging to *Azospirillum* and *Methylobacterium* were identified as keystone diazotrophic taxa for three crops and were potentially associated with microbial network stability and crop yields. Metagenomic binning recovered 58 metagenome-assembled genomes (MAGs) from the phylloplane, and the majority of them were identified as novel species (37 MAGs) and harbored genes potentially related to multiple N metabolism processes (e.g., nitrate reduction). Notably, for the first time, a high-quality MAG harboring genes involved in the complete denitrification process was recovered in the phylloplane and showed high identity to *Pseudomonas mendocina*. Overall, these findings significantly expand our understanding of ecological drivers of crop diazotrophs and provide new insights into the potential microbial N metabolism in the phyllosphere.

**IMPORTANCE:**

Plants harbor diverse nitrogen-fixing microorganisms (i.e., diazotrophic communities) in both belowground and aboveground tissues, which play a vital role in plant nitrogen supply and growth promotion. Understanding the assembly and temporal dynamics of crop diazotrophic communities is a prerequisite for harnessing them to promote plant growth. In this study, we show that the site-specific microbial species pool largely shapes the structure of diazotrophic communities in the leaves and roots of three cereal crops. We further identify keystone diazotrophic taxa in crop microbiomes and characterize potential microbial N metabolism pathways in the phyllosphere, which provides essential information for developing microbiome-based tools in future sustainable agricultural production.

## INTRODUCTION

Plants live in close association with diverse microorganisms (i.e., the plant microbiota), which are indispensable for plant fitness and performance in many aspects (1–4). Among them, symbiotic and non-symbiotic diazotrophs (nitrogen fixers, e.g., *Rhizobium* and *Azospirillum*) not only significantly contribute to plant nitrogen (N) supply but also benefit plant growth promotion *via* provisions of primary and secondary metabolites (1, 5, 6). Hence, diazotrophs are considered ideal candidates for sustaining sustainable agricultural production as plant growth-promoting rhizobacteria (PGPR) (6, 7). Increasing evidence showed that biological N-fixation (BNF) in roots, stalks, and even plant leaf surfaces and endospheres contributes significantly to the N nutrition for the host plant and the N budget in terrestrial ecosystems (5, 8–12). However, current knowledge about the diversity and ecological drivers of diazotrophs is mainly derived from soils. Given the processes of the plant microbiome assembly differ from those of the soil microbiome (e.g., the effect of host factors), it is critical to identify the fundamental ecological processes that govern the distribution and dynamics of diazotrophic communities across the soil to plant habitats. This knowledge could provide important information for harnessing the plant microbiome for sustainable agricultural production (7, 13).

Recent studies have demonstrated the immense role of multiple host and environmental factors in driving the assembly of plant-associated microbiomes (14–18). For example, it was suggested that the assembly of both bacterial and fungal communities across the soil–plant continuum is predominantly determined by plant compartment and host species, followed by environmental factors (19, 20). As for a specific compartment like the phylloplane (leaf surface) and rhizoplane, microbiomes are co-influenced by the host (e.g., plant species) and environmental (e.g., site, soil properties, and climate) factors (15, 21–24). These studies, however, mainly focused on overall bacteria and fungi, and a significant knowledge gap exists on how these multiple host and environmental factors shape the diversity and structure of diazotrophic communities along the soil–plant continuum. Given the importance of diazotrophs in plant N supply and growth promotion, theoretically, a close relationship between host and associated diazotrophs is expected (e.g., plant-specific keystone taxa) but empirical evidence is lacking. Numerous studies on soil and rhizosphere samples have suggested that site, soil pH, and fertilization are the main driving factors determining the distribution of diazotrophic communities in agriculture ecosystems (25–31). It was suggested that long-term N fertilization drastically reduced N fixation and suppressed certain diazotrophic taxa in soils (32). However, the influence of agricultural management regimes on crop-associated diazotrophs remains largely unknown.

In addition to their role in fixing N_2_, emerging evidence is showing that microbiomes might play a role in nitrification, denitrification, and N assimilation, and contribute to the decomposition of methanol and isoprene in the phyllosphere (i.e., both epiphytic and endophytic microhabitats of plant aerial parts) (15, 33–35). For example, metagenomic analyses at the contig level showed that the maize phyllosphere microbiome harbors diverse N-cycling functional genes (15). Phyllosphere microorganisms are expected to play critical roles in atmospheric N cycling since the phyllosphere acts as the plant-atmosphere interface and is usually exposed to multiple environmental changes like N deposition (36, 37). However, a systematic understanding and genome-based evidence on the role of phyllosphere microbiomes in N cycling in agroecosystems is still scarce.

In this study, we used amplicon sequencing of the *nif*H gene to examine diazotrophic communities across soils (bulk and rhizosphere soils), plastic leaf (representing local air environment), and multiple compartments (rhizoplane, root endosphere, phylloplane, and leaf endosphere) of three crops (maize, wheat, and barley) under four fertilization regimes in two major agricultural areas of China (Fig. S1). We then explored the N-cycling pathways of phylloplane microbiomes *via* metagenomic binning. We aimed to (1) determine the distribution pattern of diazotrophic communities along the soil–plant continuum of different crops and across different plant developmental stages (2); identify dominant diazotrophic taxa of crop microbiomes and their potential ecological role in microbial co-occurrence network and plant performance; and (3) examine the potential microbial N metabolism pathways in phylloplane microbiomes in agricultural ecosystem, based on metagenome-assembled genomes. We hypothesized that (1) both host selection, driven by crop species and developmental stage, and site-specific microbial species pool will influence equally the assembly of crop-associated diazotrophic communities (2); some specific diazotrophic taxa will be associated with plant selection effects and may play key roles in microbial co-occurrence network and crop production; and (3) phylloplane microbiomes may possess ample N metabolism pathways, as they are typically exposed to atmospheric N cycling.

## RESULTS

### Distribution pattern and drivers of diazotrophic communities across soil and plant compartments

**Fig 1:**
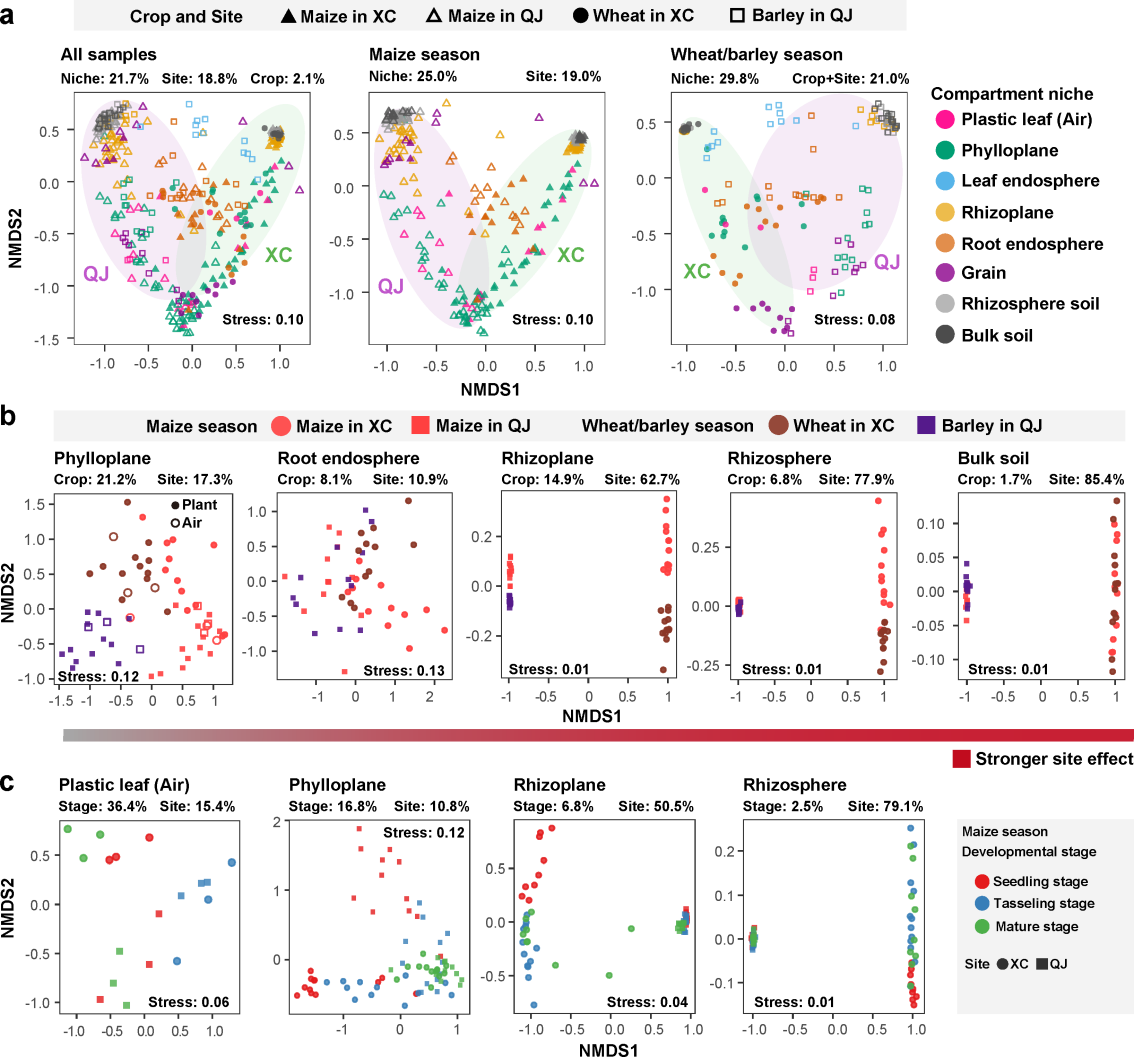
Distribution and temporal dynamics of crop-associated diazotrophic communities. (**a**) Non-metric multi-dimensional (NMDS) ordinations depicting the distribution patterns of diazotrophic communities along the soil–plant continuum from all samples (*n* = 426), maize samples (*n* = 270), and wheat/barley samples (*n* = 156). Ellipses include 80% of samples from each site. (**b**) NMDS ordinations showing the effects of crop species and site on diazotrophic communities in each compartment (phylloplane: *n* = 60; other niches: *n* = 48). The gradient bar chart represents the influence of the site on the diazotrophic communities, with intensified red indicating an increased influence of the site on the community. (**c**) NMDS ordinations of maize diazotrophic communities across three plant developmental stages in the phylloplane, rhizoplane, and rhizosphere (phylloplane: *n* = 144; other niches: *n* = 54). The relative contribution of host and environmental factors on community dissimilarity was tested with PERMANOVA. “Niche” represents the effect of compartment niche, “Crop” represents the effect of crop species, “Site” represents the effect of site, and “Stage” represents the effect of developmental stage. “XC” represents the site “Xuchang,” “QJ” represents the site “Qujing.”

**Fig 2:**
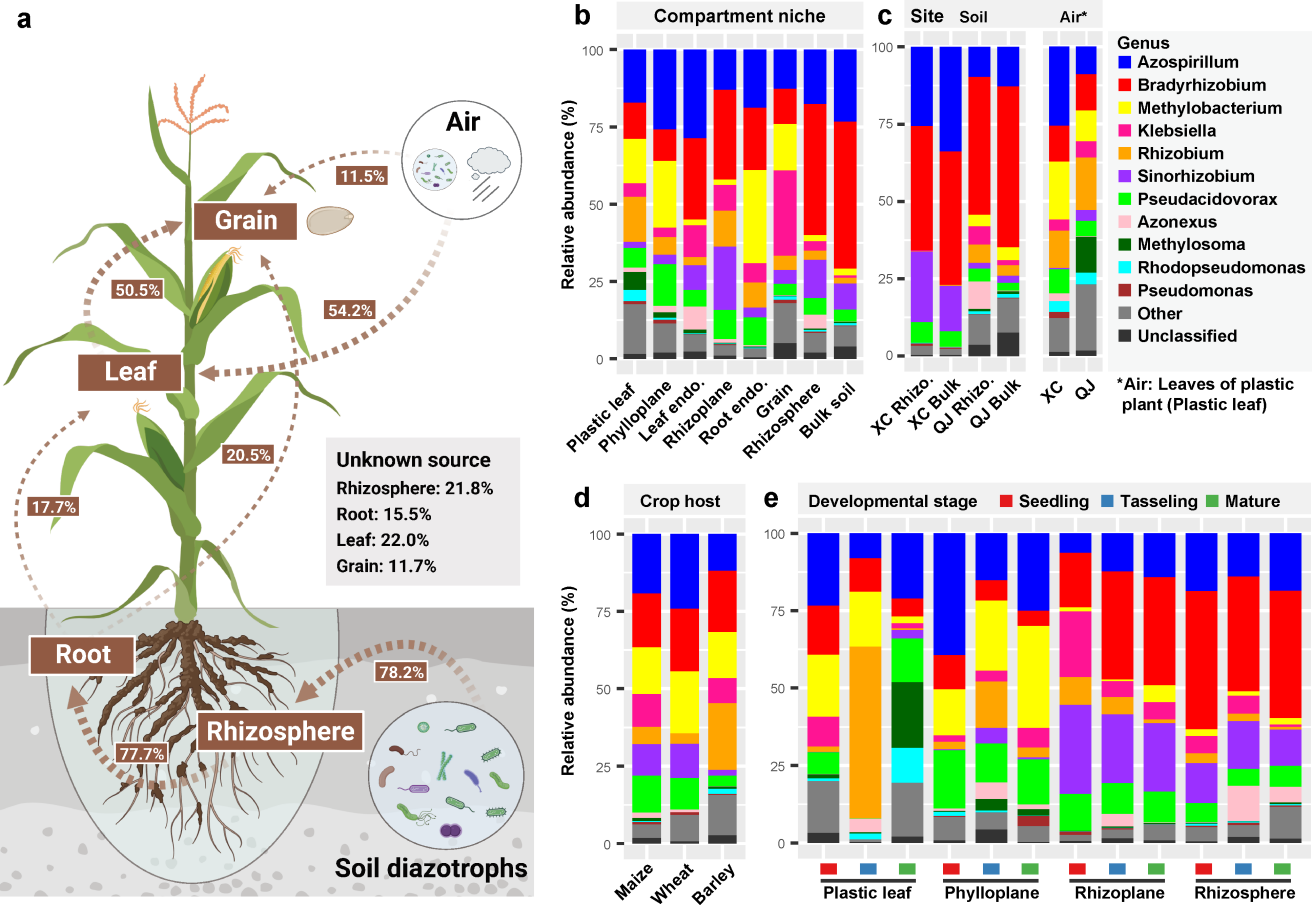
Potential sources and taxonomic composition of crop-associated diazotrophic communities. (**a**) Source-tracking analysis showing potential sources of crop-associated diazotrophic communities based on maize samples collected in 2017 (*n* = 270). Taxonomic composition of diazotrophic communities among (**b**) different compartment niches, (**c**) two sites, (**d**) three crop hosts, and (**e**) three developmental stages. The genera with the relative abundance <1% are grouped into “Other.” “endo.” represents “endosphere.” “rhizo.” represents “rhizosphere soil.” “XC” represents the site “Xuchang,” “QJ” represents the site “Qujing.”

The high-throughput sequencing of *nif*H gene based on 426 samples, which collected from seven plant and soil compartments under four fertilization treatments and the leaf of artificial plants in two geographically distant sites, produced a total of 18,750 diazotrophic operational taxonomic units (OTUs), primarily belonging to genera *Bradyrhizobium* (10,535 OTUs), *Azospirillum* (3,063), *Pseudacidovorax* (1,301), *Sinorhizobium* (547), and *Methylobacterium* (391). Our PERMANOVA analyses further showed that the variation in the diazotrophic community across the soil–plant continuum was mainly explained by compartment (*R*^2^ = 21.7%, *P* < 0.001) and site (*R*^2^ = 18.8%, *P* < 0.001), with slight influence from crop species (*R*^2^ = 2.1%, *P* < 0.001), plant developmental stage (*R*^2^ = 4.4%, *P* < 0.001), and fertilization practice (*R*^2^ = 2.6%, *P* < 0.001) (Fig. 1a; Table S1). When analyzing different compartments separately, the site played an increasing effect in shaping diazotrophic communities across the root endosphere (*R*^2^ = 10.9%, *P* = 0.009), phylloplane (*R*^2^ = 17.3%, *P* < 0.001), rhizoplane (*R*^2^ = 62.7%, *P* < 0.001), rhizosphere (*R*^2^ = 77.9%, *P* < 0.001), and bulk soil (*R*^2^ = 85.4%, *P* < 0.001), indicating a stronger site effect on the diazotrophic community in soils than in plant compartments (Fig. 1b; Table S1). Meanwhile, the diazotrophic community in the plastic leaf (i.e., airborne diazotrophs) was significantly different from soil diazotrophs (*R*^2^ = 22.8%, *P* < 0.001) and differed between two sites (*R*^2^ = 16.9%, *P* < 0.001), indicating that there are distinct soil and air microbial species pools between two sites (Fig. 1; Table S1). Source-tracking analyses further showed that soil diazotrophs accounted for 78.2% of the sources contributing to rhizosphere diazotrophic communities, and 77.7% of root and 54.2% of leaf diazotrophic communities were derived from rhizosphere and airborne diazotrophs, respectively (Fig. 2a). These results revealed the pivotal role of the local microbial species pools in structuring plant-associated diazotrophic communities.

We then explored the temporal dynamics of the diazotrophic community. The diazotrophic community in the phylloplane (*R*^2^ = 16.8%, *P* < 0.001) was more sensitive to the plant developmental stage than those in the rhizoplane (*R*^2^ = 6.8%, *P* = 0.03) and rhizosphere (*R*^2^ = 2.5%, *P* = 0.015) (Fig. 1c; Table S1). Sampling time (*R*^2^ = 36.4%, *P* < 0.001) also showed a significant effect on the diazotrophic community in the plastic leaf (Fig. 1c; Table S1). For the alpha-diversity, the diazotrophic Shannon index was highest in bulk and rhizosphere soils, and followed by rhizoplane, but showed no significant difference among the three crops (Fig. S2). Sampling time had a significant effect on the Shannon index of diazotrophic communities in both air and rhizoplane samples (Fig. S2).

To further explore the response of diazotrophic communities in each compartment to four fertilization practices, that is, no N fertilizer (Control), 80% N fertilizer (80%N), biochar treatment (80%NSB), and straw treatment (80%NS), sub-dataset of each compartment were subjected to PERMANOVA analyses. It showed that fertilization practice explained 33%–51% of community variation in each compartment at two sites (Fig. S3a and b). Particularly, biochar treatment displayed a significant effect on diazotrophic community composition in most plant and soil compartments (Fig. S3a and b), and significantly increased diazotrophic Shannon index in both rhizosphere and bulk soils at the site XC, compared with its counterpart treatment (80%NS) (Fig. S2e).

### Taxonomic composition and dominant taxa of crop-associated diazotrophs

Taxonomic classification showed that diazotrophic community largely varied between plant and soil habitats, with more abundant *Methylobacterium* in plant compartments (leaves, roots, and grain) (~15.3% vs ~2.0% in soils) while more abundant *Bradyrhizobium* in soils (~44.3% vs ~18.7% in plants) (Fig. 2b; Fig. S3c). As for two sites (including soil and air samples), site XC had a higher proportion of *Azospirillum* and *Sinorhizobium* while site QJ harbored more abundant *Methylobacterium*, *Methylosoma*, and *Rhodopseudomonas* (Fig. 2c; Fig. S3d). Among the three crops (only includes plant samples), more abundant *Rhizobium* was observed in barley while more abundant *Pseudacidovorax* was found in maize or wheat (Fig. 2d; Fig. S3e). The linear mixed model analysis suggested that the plant developmental stage had the strongest effect on *Rhizobium* (*F* = 9.7, *P* = 9.7e–5), which was more abundant at the tasseling stage than at other stages in both plastic leaf and maize phylloplane (Fig. 2e; Fig. S3f). The differential abundance analysis showed that some members within *Bradyrhizobium* were significantly enriched in 80%NSB treatment in soils in both XC (OTU9001 and OTU19639) and QJ (OTU6065) sites (Fig. S4).

**Fig 3:**
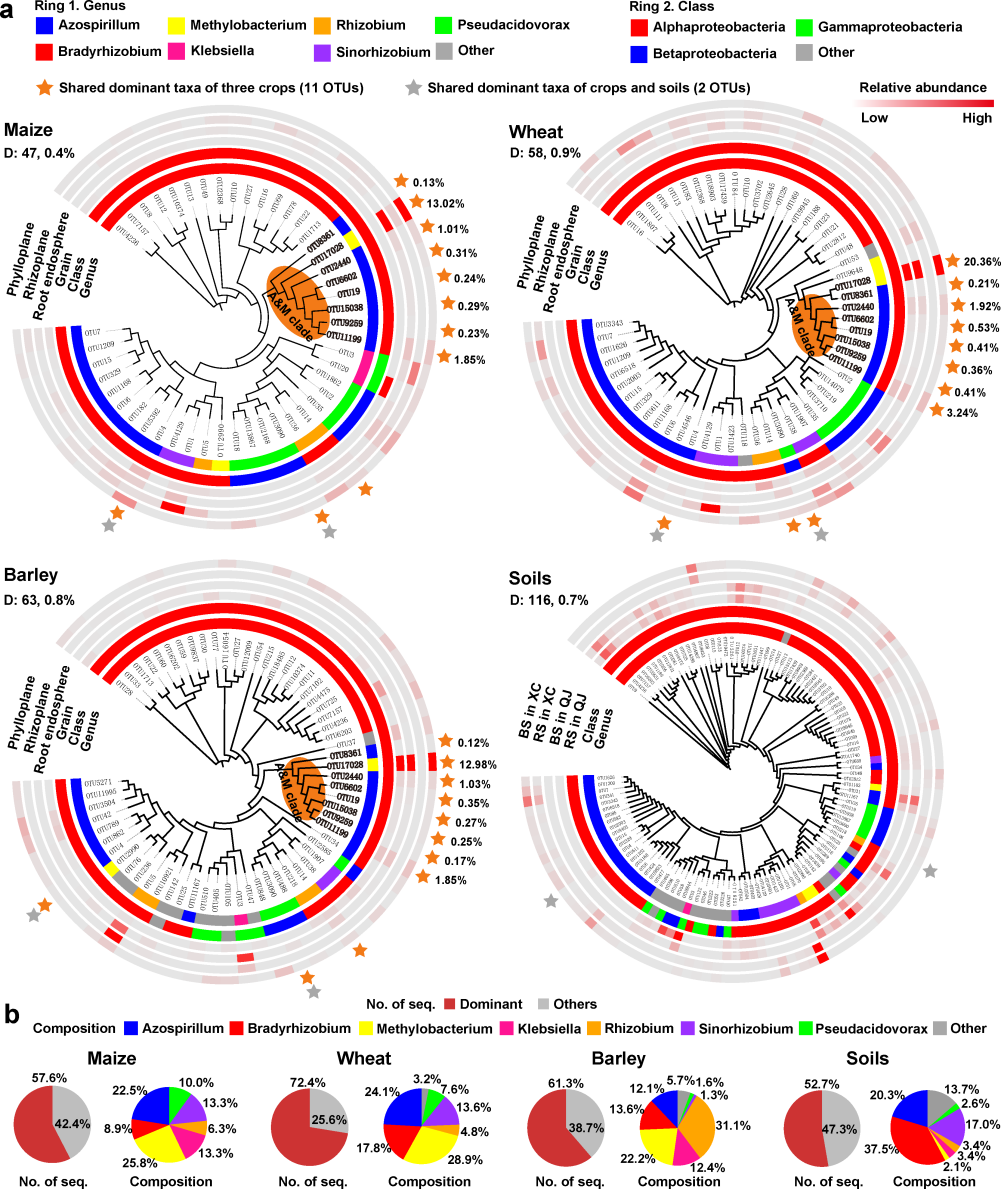
Taxonomic composition and distribution patterns of dominant sub-community in soils and plant compartments. (**a**) Identification of dominant taxa for three crop hosts (only considering leaf, root, and grain compartments; maize: *n* = 168, wheat: *n* = 45, barley: *n* = 45) and soils (include rhizosphere and bulk soils, *n* = 132). The numbers above the A&M clade taxa represent its average relative abundance in plant compartments. (**b**) Pie plot showing the proportion and community composition of the dominant sub-community in each crop and soil. The dominant taxa of diazotrophic communities were defined as OTUs with an average relative abundance ≥0.1% and present in at least one-third of the samples evaluated. The genera with the relative abundance <1% are grouped into “Other.”

To further identify the keystone diazotrophic taxa across the soil–plant continuum, we defined the dominant taxa as the OTUs with a relative abundance ≥0.1% and present in at least one-third of samples evaluated and explored the composition of dominant sub-communities. It showed that crop diazotrophic communities were dominated by a few taxa (0.4%–0.9% of phylotypes) mainly belonging to *Methylobacterium*, *Azospirillum*, *Bradyrhizobium*, and *Rhizobium*, which accounted for 57%–72% of the total sequences in each crop (Fig. 3a and b). By contrast, soil diazotrophic communities were dominated by a few taxa mainly belonging to *Bradyrhizobium* and *Azospirillum* (Fig. 3b). Further analysis showed that eight dominant taxa belonging to *Azospirillum* (7 OTUs) and *Methylobacterium* (1 OTU) were commonly present in the three crops with close phylogenetic relationships and high relative abundance (17%–27%) in each crop, but were absent in soils, and thus were defined as crop-specific taxa (termed the A&M clade) (Fig. 3a). Mantel test based on maize season samples indicated that soil pH, NO_3_^-^-N, and DOC were important drivers of the diazotrophic community and that maize dominant sub-community in the rhizoplane and rhizosphere had greater correlations with rhizosphere nitrogenase activity than whole and non-dominant sub-communities across three plant developmental stages (Table S2). Random forest modeling analyses indicated that the relative abundance of the A&M clade taxa was the most important predictor for crop yield in each crop species (Fig. S5a through c).

### Co-occurrence networks of crop-associated diazotrophic communities

**Fig 4:**
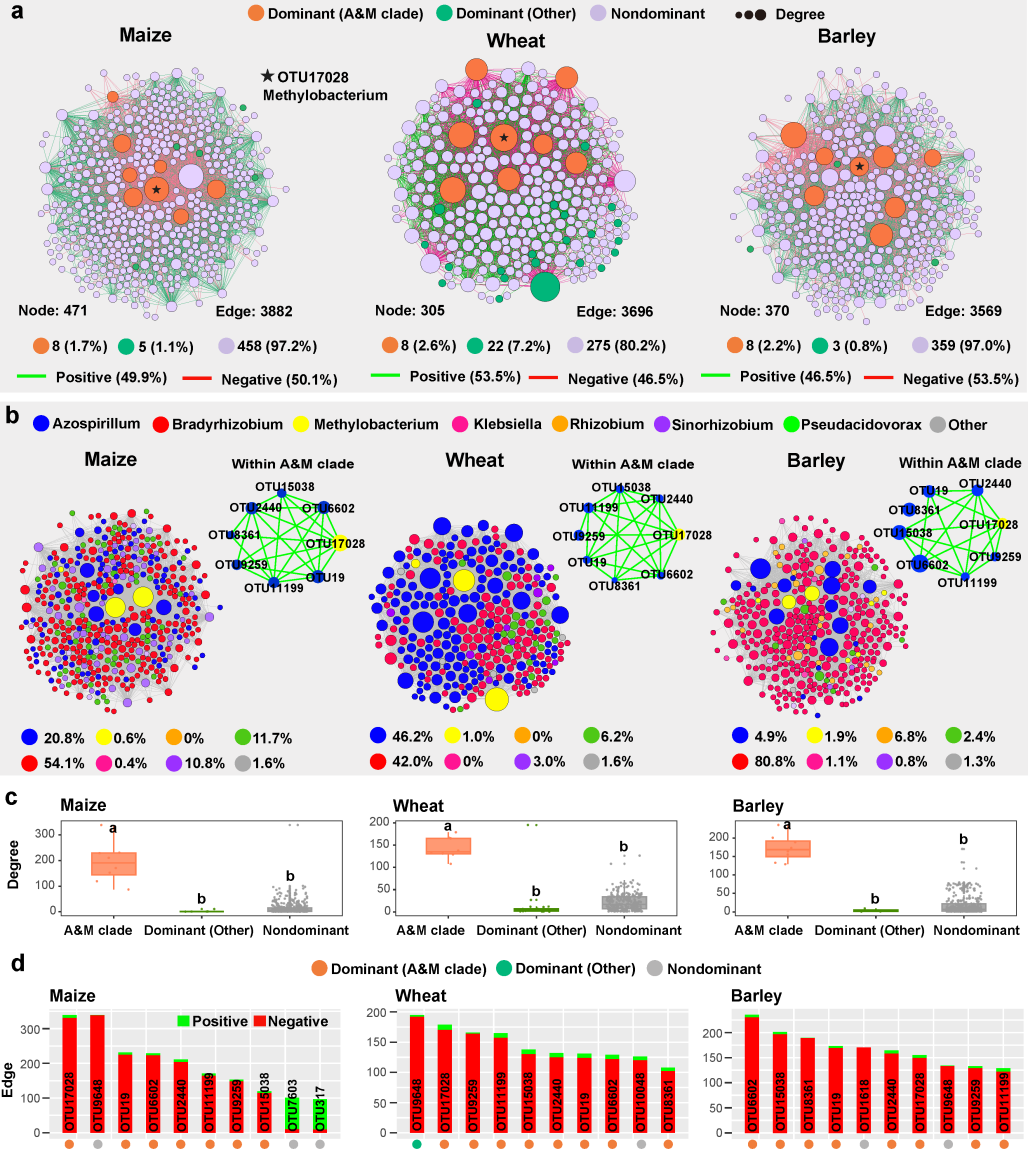
Co-occurrence networks of crop-associated diazotrophic communities. Co-occurrence network analysis showing microbial network patterns of three crop hosts (only considering leaf, root, and grain compartments; maize: *n* = 168, wheat: *n* = 45, barley: *n* = 45). The nodes of the network are colored according to (**a**) different sub-communities (i.e., dominant and non-dominant taxa) and (**b**) diazotrophic genus. The sizes of the nodes are according to the degree of connection, and the edges color represents positive (green) and negative (red) correlations. (**c**) Comparison of node-level topological features (i.e., degree) among different sub-communities (i.e., dominant and no-dominant taxa). (**d**) Identification of 10 hubs with the top degree in the network from each crop.

Network analysis showed that the co-occurrence patterns of the diazotrophic community differed among the three crops, with more nodes belonging to *Azospirillum* and *Sinorhizobium* in maize and wheat, and *Bradyrhizobium* and *Rhizobium* in barley (Fig. 4). More importantly, all eight A&M clade taxa had significantly higher network degree than any other dominant and non-dominant taxa (Fig. 4a and c). Among the top 10 hubs with the highest degree in the network of each crop, 7–8 hubs belonged to the A&M clade (Fig. 4d), among which OTU17082 (*Methylobacterium*) was identified as the most important network hubs for both maize and wheat (Fig. 4b and d). Interestingly, the hubs belonging to A&M clade taxa showed a positive correlation between them but mainly showed a negative correlation with other diazotrophic taxa in the network of three crops (Fig. 4b and d). In addition, diazotrophic co-occurrence patterns differed among different compartments, with more nodes belonging to *Azospirillum* in the phylloplane (both plant and plastic) (Fig. S6).

### Functional profiles of MAGs in phylloplane microbiomes

**Fig 5:**
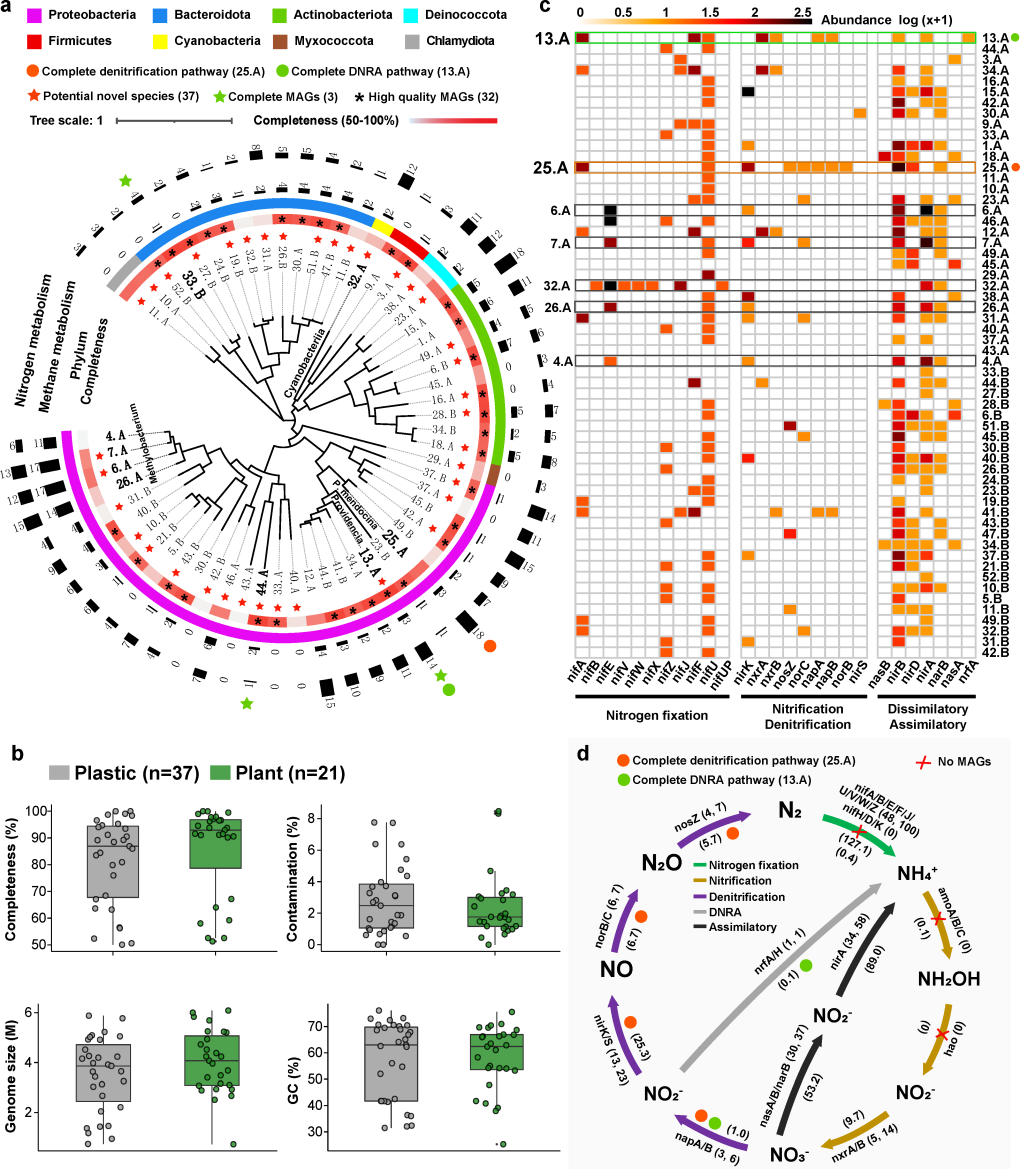
Phylogenetic tree and nitrogen-cycling pathways of the MAGs recovered from phylloplane microbiomes. (**a**) Phylogenetic tree of 58 bacterial MAGs recovered from phylloplane microbiomes, including 31 MAGs recovered from the plastic leaf (named “.A”) and 27 MAGs from the maize phylloplane (“.B”), respectively. (**b**) Box plot showing the quality information (e.g., completeness and contamination) of all MAGs. (**c**) Heat map exhibiting the abundance of functional genes (based on KEGG Orthology) related to nitrogen cycling processes (e.g., nitrogen fixation, nitrification, and denitrification) in 58 MAGs. Detailed information on the 58 MAGs, including completeness, contamination, genome sizes, and taxonomic classification, can be found in the supplementary materials shared on Figshare (https://figshare.com/s/24f2a51568915f1138d6). (**d**) Nitrogen cycling pathways recovered based on the functional genes in 58 MAGs from the plastic and maize phylloplanes. The numbers in the brackets after the gene name represent the number of genomes identified and the total gene abundance, respectively. The number in the inner ring represents the average gene abundance (reads per million, RPM) based on the contig-level analysis.

Metagenomic binning successfully recovered 31 bacterial MAGs from the artificial leaf (named “*.A”) and 27 MAGs from the maize phylloplane (“*.B”), among which 32 high-quality MAGs had a completeness of >90% (contamination <5%) and three had a completeness of 100% (nearly complete MAGs) (Fig. 5a and b). These MAGs were mainly assigned into the phylum *Proteobacteria* (29 MAGs), *Bacteroidota* (12), and *Actinobacteriota* (9), among which ~63% of MAGs (37 out of 58 MAGs) were identified as potential novel species (Fig. 5a). Furthermore, the most abundant functional genes (top 20) possessed by these 58 MAGs were related to ABC transporters (e.g., genes related to iron, sugar, and amino acid transportation) and methyl-accepting chemotaxis protein (i.e., *mcp*) (Fig. S7a).

Among all the MAGs, most harbored genes related to nitrate reduction (e.g., *nar*B and *nas*A/B, 30 MAGs) and denitrification (*nir*K/S, 13 MAGs) processes. Furthermore, 48 MAGs possessed N fixation-related genes (e.g., *nif*A/B/E/F/J), which were associated not only with electron transfer and cofactors but also with energy metabolism and biosynthesis of secondary metabolites. However, none of them possessed genes encoding nitrogenase (i.e., *nif*H/D/K) (Fig. 5c and d). In addition, one MAG (32.A) affiliated with *Cyanobacteria* harbored diverse N-fixation-related genes including *nif*B*/*E/V/W/X/J and genes involved in nitrate assimilatory (*nir*A and *nar*B) and photosystem (*psa*A). Similar to the amplicon sequencing results, four MAGs (4.A, 6.A, 7.A, and 26.A) were identified as *Methylobacterium* spp. and possessed more abundant genes involved in N and methane metabolisms (Fig. 5a). The two MAGs (4.A and 7.A) consistently contained genes related to denitrification (*nir*K), nitrate assimilation (*nir*A and *nar*B), and methanol oxidation genes (*mdh*1/2) (Fig. 5c; Fig. S7b). Surprisingly, a high-quality MAG 25.A (completeness 89.3%, contamination 5.4%) identified as *Pseudomonas mendocina* (~4.9M, ANI 98.4%) possessed multiple genes involved in complete denitrification pathway (i.e., *nap*A/B, *nir*K, *nor*B/C, and *nos*Z). Furthermore, it also possessed genes related to aerobic respiration (high O_2_*cox*A/*cyo*A, low O_2_*cco*N/*cyd*A), fumarate reductase (*frd*A), lactate dehydrogenase (*ldh*), and formate dehydrogenase (*fdo*G) (Fig. 5c and d; Fig. S7b). Another complete MAG 13.A (potential novel species) with high identity similarity with *Providencia* spp. possessed genes related to complete dissimilatory nitrate reduction to ammonium (DNRA) (*nap*A/B and *nrf*A), aerobic (*cox*A/*cyo*A and *cyd*A) and anaerobic (*hya*B) respirations, and photosystem (*psa*A and *psb*A) (Fig. 5c and d; Fig. S7b). In addition, non-ribosomal peptide synthetase (NRPS) gene clusters (~13 kb in length) were identified from MAG 25.A and a gene clusters (~31 kb) involved in thiopeptide were identified from MAG 13.A. Though these MAGs (e.g., 25.A, 13.A, 4.A, and 7.A) were retrieved from the plastic leaf, their relative abundances were relatively lower in comparison to the maize phylloplane (Fig. S7c).

## DISCUSSION

### Compartment and site dominate over host species in determining the diazotrophic community across the soil–plant continuum

Understanding the ecological drivers of crop-associated diazotrophic communities is integral to harness diazotrophs for sustainable agricultural production. To reach this target, we examined diazotrophic communities across the soil–plant continuum of three crops under different fertilization practices in two geographically distant soils and incorporated artificial plants as background controls. Our study demonstrated that compartment and site are the primary drivers shaping the assembly of crop diazotrophic communities across the soil–plant continuum. As for the bulk and rhizosphere soils, diazotrophic communities were largely determined by site (77%–85%). These findings were consistent with what has been observed previously in bacterial and fungal communities (19, 20, 38, 39). Surprisingly, we found that the site (10%–62%) plays a more important role in determining diazotrophic community in most plant compartments than crop species (8%–21%) and plant developmental stage (6%–16%). More specifically, maize diazotrophs in the root endosphere from site QJ were more closely assembled with barley diazotrophs from the same site, rather than with same the crop from site XC (Fig. 1b). This result contrasts with our previous observation showing that crop species (bacteria ~52%, fungi ~41%) dominated over site (bacteria ~20%, fungi ~15%) in determining the assembly of bacterial and fungal communities in plant compartments (19, 20). The diazotrophic community in the artificial leaf (i.e., aerobic background) differed between the two sites and was more sensitive to sampling time than those of the maize, indicating the important role of season-dependent climatic factors on leaf diazotrophs. These results collectively reveal that, in comparison to the overall bacterial and fungal communities, the diazotrophic community, as a functional group within bacteria, is more sensitive to site-dependent environmental factors but more stable to host effect caused by crop species and developmental stage.

**Fig 6:**
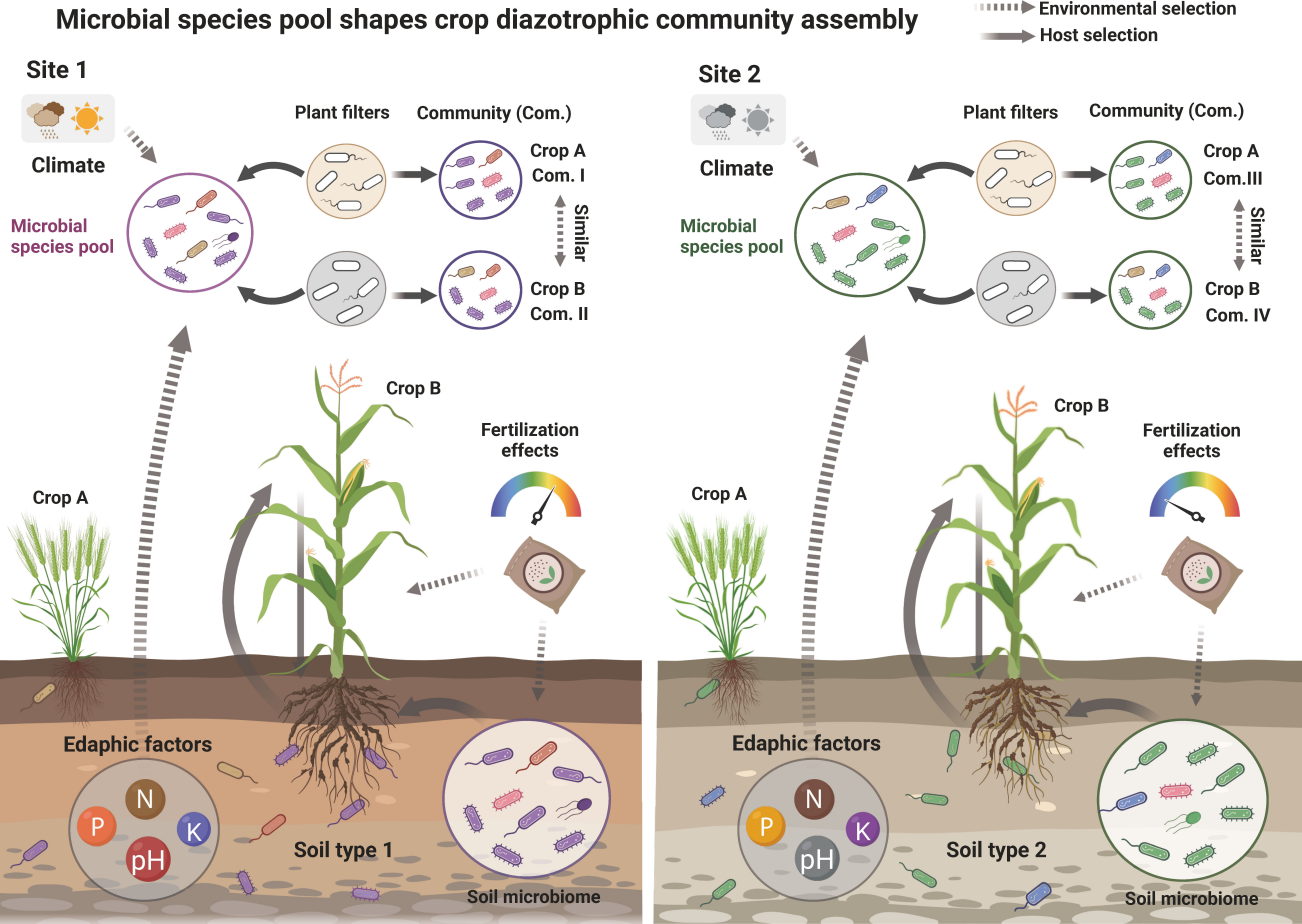
The conceptual models of crop diazotrophic community assembly. Crop-associated diazotrophic communities are largely shaped by environmental factors including climate (e.g., temperature and precipitation), edaphic factors (e.g., soil type, pH, and nutrients), and soil fertilization practices. These environmental factors define not only plant growth and development but also the microbial species pool and soil–plant interactions, which, in turn, shape crop diazotrophic community assembly.

Thus, we proposed a conceptual model to explain the ecological mechanisms that govern the assembly of crop-associated diazotrophic communities under different environmental conditions (Fig. 6). Our study demonstrated that the influence of site on diazotrophic communities outweighs that of crop species and developmental stage in bulk/rhizosphere soils and most plant compartments. Further analyses indicated that soil and airborne diazotrophs were important sources of plant diazotrophs. Together, these results revealed that the local microbial species pool developed under strong site effects plays an important role in shaping diazotrophic communities across the soil–plant continuum. Our results showed that soil pH and nutrients including available N and DOC are primary determinants affecting the assembly of soil diazotrophic communities. Site-dependent climatic factors (e.g., temperature and precipitation) and agronomic regimes (e.g., fertilization habits) also play key roles in structuring the microbial species pool of diazotrophs in soils and atmosphere, which further exerts influence on the diazotrophic communities in plant compartments (Fig. 6). On the other hand, the site-dependent edaphic and climatic factors also define plant growth and development (2, 40), which might, in turn, shape crop diazotrophic community assembly. The sites QJ and XC had distinct climates (subtropical monsoon vs temperate monsoon), soil properties (acidic red soil vs alkaline Fluvo-aquic soil), and different atmospheric deposition (25, 41), which facilitate the differentiation of microbial species pool between two sites and profoundly affected the assembly of crop-associated diazotrophic communities (Fig. 6).

Furthermore, many studies have reported that different agricultural management regimes (e.g., fertilization practice) can significantly affect the diversity and structure of soil diazotrophic communities (29, 32, 42). Here we provide new evidence that soil amendment practices like biochar addition can not only influence the diazotrophic community in soils (enhancing diversity, changing the community structures, and enriching *Bradyrhizobium*) but also affect the diazotrophic community in plant compartments. These results concur with previous findings on the positive impacts of biochar on soil microbiomes and fertility (43, 44) and extend our understanding of crop microbiomes. The biochar amendment may exert its influence on crop diazotrophs directly by affecting the soil microbial species pool, or indirectly by affecting plant growth and development (45). These findings implied the possibility of manipulating the diazotrophic community for sustainable crop production and thus has significant implications for agricultural practices.

### Keystone diazotrophic taxa in soil and crop microbiomes

Our study further showed that the crop diazotrophic community was dominated by a few phylotypes which were mainly affiliated with *Methylobacterium*, *Azospirillum*, *Bradyrhizobium*, and *Rhizobium*. Coincidently, previous studies suggest that bacterial and fungal communities in soils (46) and plant compartments (19, 20) were dominated by a few dominant taxa. More intriguingly, eight dominant taxa (A&M clade) comprised of *Azospirillum* and *Methylobacterium* were identified as keystone diazotrophic taxa in three crops and played a potential role in predicting crop yield in this study. Similarly, a recent study suggested that the diversity of keystone phylotypes in soil microbial communities had positive associations with crop production (27). We further found that the network hubs belonging to the A&M clade had higher proportions of positive edges between them (i.e., positive associations). Coincidently, a previous study by Madhaiyan et al. found that the co-inoculation of *Methylobacterium oryzae* and *Azospirillum brasilense* displayed a significantly positive effect on the growth and nutrient uptake of various crops, including tomato, red pepper, and rice (47). These together with our findings suggested that *Azospirillum* and *Methylobacterium* might synergistically function in plant compartments. However, we must acknowledge that the positive correlations between A&M clade taxa observed in the network may be attributable to similar selection factors, rather than actual synergistic interactions. The real microbial interactions within the A&M clade and among different groups need further investigation. The observed diversity and dominance of diazotrophic communities across various crops further revealed the functional redundancy of plant microbiomes, which might be a strategy of the microbiome to warrant a given function being maintained in a changing environment.

Furthermore, these A&M clade taxa were significantly enriched in plant compartments in comparison to bulk and rhizosphere soils, suggesting their preference for plant compartments. Some members of *Methylobacterium* were frequently identified as dominant taxa in the phyllosphere of various plants (15, 48) and demonstrated to be able to fix N_2_ and promote plant growth (49). Our metagenomic analyses further showed that two MAGs belonging to *Methylobacterium* in the phylloplane contained genes involved in N and methane metabolisms, further confirming their potential function in the phyllosphere. The members of *Azospirillum* are capable of colonizing a wide range of habitats like the phyllosphere and rhizosphere and play a key role in modulating host fitness (50, 51), which may explain their dominant distribution in various plant compartments. These results suggest that *Methylobacterium* and *Azospirillum* may play vital ecological roles in plant performance while their true function needs further validation through synthetic community experiments.

We further found that *Rhizobium* was more abundant in barley than in maize or wheat, suggesting that the barley species may preferentially enrich members of *Rhizobium*. Except for N fixation with leguminous plants, many members of *Rhizobium* are supposed to be important PGPR which cannot form nodules with plants but are capable of promoting plant growth *via* secreting phytohormones and siderophores, inducing plant immunity response and fitness to biotic and abiotic stresses on non-legume plants (52–54). As for soils, dominant diazotrophic sub-communities were dominated by *Bradyrhizobium*, which was further enriched by biochar amendment (80%NSB). *Bradyrhizobium* was consistently recognized as the dominant diazotrophic taxa across diverse ecosystems and is believed to play key roles in active N fixation in soils (27, 55, 56). The identification of these keystone diazotrophic taxa provides critical information for engineering synthetic communities (SynComs) and manipulating crop–diazotrophs interactions for future sustainable agricultural production. On the other hand, we must acknowledge that the identification of these dominant and keystone taxa might be influenced by the bias of primers used in this study (57–59), as previous studies have indicated that the use of primer PolF/PolR for *nif*H gene amplification may result in a lower diversity and a preference for amplifying *Proteobacteria*, *Firmicutes*, and *Actinobacteria* (57, 59). More intensive research employing different primers is necessary to better profile soil- and plant-associated diazotrophic communities in the future.

### The potential microbial N metabolisms and function in the phylloplane

Phyllosphere microbiomes can provide benefits to the plant in multiple aspects (60–62), but our knowledge of microbial N metabolism in the phyllosphere is largely limited. Our metagenomic binning for phylloplane microbiomes suggested that 48 of 58 retrieved MAGs harbored genes related to electron transfer (e.g., *nif*F/J) and cofactor (e.g., *nif*U/V/B) in N fixation processes. However, none of the 58 MAGs possessed genes encoding nitrogenase (i.e., *nif*H/D/K), and some dominant diazotrophic taxa identified by the amplicon sequencing, such as *Azospirillum*, *Bradyrhizobium*, and *Rhizobium*, were not recovered in metagenomic binning (i.e., not within 58 MAGs). These might have been caused by the limited sequencing depth in this study and the low MAG recovery rate due to the low abundance of diazotrophs in the phylloplane. The activity and N fixation function of the diazotrophic community in the phylloplane therefore need further exploration based on deeper sequencing analysis or more powerful techniques such as ^15^N tracing or stable isotope probe (SIP). Remarkably, a high-quality MAG 25.A with high identity similarity to *P. mendocina* possessed genes involved in a complete denitrification pathway (i.e., covering all four denitrification processes from NO_3_^−^→NO_2_^−^→NO→N_2_O→N_2_). Though biological nitrification and N_2_O production *via* ammonia-oxidizing bacteria (AOB)-mediated nitrification in the phyllosphere have been previously suggested in forests (34) and pasture (63), to the best of our knowledge, this is the first time that microbial genomes with complete denitrification-related genes were identified in the phylloplane. Although the genome contains genes indicating potential for certain functions, this does not necessarily mean that the microbe expresses these functions in the phylloplane. This observation suggested that phylloplane microbiomes possess diverse metabolic pathways that confer their plasticity to adapt to different environments and conditions. Their actual ecological function in the phylloplane requires further experimental validation. On the other hand, the potential denitrification at the site QJ could be explained by the fact that excessive atmospheric N deposition in the form of NOx (34, 64). N deposition is one of the major N sources for agricultural areas in southwest China close to the QJ site (~20 kg N ha^−1^ yr^−1^) (64, 65). High denitrification ability under aerobic condition had been reported in *P. mendocina* from sewage treatment and other members of *Pseudomonas* (66–68). These findings, together with the detection of genes related to aerobic respiration, fumarate reductase, lactate dehydrogenase, formate dehydrogenase, and non-ribosomal peptide synthetase (NRPS) in MAG 25.A, suggest that *P. mendocina* has the potential for diverse metabolic pathways.

We further identified a complete MAG 13.A that was highly identical to *Providencia* spp. and possessed genes related to complete DNRA pathways and photosystem. The intermediate product of denitrification and DNRA processes, nitric oxide (NO), is an important gaseous signaling molecule that regulates multiple physiological processes associated with plant development and stress responses, such as stomatal closure and photosynthesis regulation (69, 70). Therefore, nitrate reduction-related functional genes and bacterial taxa retrieved in this study might be closely associated with plant signaling in the phyllosphere, which deserves exploration in further study. Overall, this study significantly advances our knowledge of the microbial N metabolism and the potential ecological function of phyllosphere microbiomes.

### Conclusions

This study provides the first comprehensive empirical understanding of the diversity, composition, and keystone taxa of diazotrophic communities across multiple soil and plant compartments of three cereal crops. Our results demonstrated that the assembly of diazotrophic communities across the soil–plant continuum is determined largely by compartment and site, followed by crop species and developmental stage. We further found that the effect of the site on diazotrophic communities outweighed those of crop species and developmental stage in most of the soil and plant compartments and that soil and airborne diazotrophs were important sources of plant diazotrophs. These findings highlight that the site-specific microbial species pool developed under long-term site effect plays a primary role in shaping the diazotrophic communities across the soil–plant continuum. Our study further identified eight dominant diazotrophic taxa (belonging to *Azospirillum* and *Methylobacterium*) as keystone taxa in crop diazotrophs, which might be responsible for regulating microbial network stability and acting as key N fixers and plant growth-promoting bacteria in crops.

Furthermore, 58 bacterial MAGs were recovered from plastic and maize phylloplane microbiomes and the majority of them were identified as novel species (37 MAGs) and harbored genes related to N metabolism (e.g., nitrate reduction) processes. We further identified two high-quality MAGs (close to *P. mendocina* and *Providencia* spp.) harboring genes involved in the complete denitrification pathway and DNRA pathway, respectively. These findings significantly advanced our knowledge of the assembly of diazotrophic communities across soil and plant compartments and provided an important direction for harnessing the plant microbiome for sustainable development goals.

## MATERIALS AND METHODS

### Experimental design and sampling

The field experiments with seven fertilization treatments were established in Xuchang, Henan province (XC, the alkaline Fluvo-aquic soil located in northern China), and Qujing, Yunnan province (QJ, the acidic red soil located in southwest China) in 2016 as previously described (19, 20). To evaluate the distribution pattern of diazotrophic communities along the soil–plant continuum of different crops and across different plant developmental stages, four treatments including (T1) Control (zero N fertilizer); (T2) 80%N (20% N reduction based on local farmers’ N rate); (T3) 80%NS (80%N treatment plus straw covering); and (T4) 80%NSB (80%NS treatment plus biochar addition) were involved in this study. For each crop, samples from five compartments (including leaf, grain, root, rhizosphere, and bulk soil) were collected following our previous method (15, 19). For maize season, sample collections were performed in June, August, and September of 2017, corresponding to maize seedling, tasseling, and mature stage, respectively (*n* = 282). Sampling for the wheat/barley season was performed once at the grain filling stage in April 2018 (*n* = 162) (Fig. S1a and b). Artificial plants made of plastic material were planted as “background controls” in the field, and plastic leaves were sampled at the same time with soil and plant sample collection (*n* = 24) (15). Thus, a total of 468 samples were retrieved for this study, of which maize season samples in 2017 were used to examine bacterial and fungal communities in our previous studies (15, 19, 20). Soil physicochemical parameters (e.g., pH, NH_4_^+^-N, and NO_3_^-^-N) were measured according to previous protocols (15, 20, 71, 72). Soil nitrogenase activity was measured using the acetylene reduction method (15, 20). The crop yield data were collected based on the field harvest (15).

### DNA extraction and *nif*H gene amplification

Rhizosphere and bulk soil DNA were extracted from 0.4 g soil using the PowerSoil DNA Isolation Kit (MO BIO Laboratories, Carlsbad, CA, USA). Epiphytic and endophytic DNA from leaves (10–15 g) and roots (3–5 g) of maize and wheat/barley, and microbial DNA from the plastic leaf and plant grain samples were extracted using the PowerSoil DNA Isolation Kit as described previously (15, 19). The *nif*H genes were amplified using the primer pairs PolF/PolR (41, 73). In total, 426 DNA samples from soils, multiple plant compartments, and plastic leaves were successfully amplified (42 DNA samples failed to be amplified, Fig. S1). More detailed descriptions on the field trial and the *nif*H gene amplification are available in Supplementary Information (Method S1).

### Bioinformatic analysis for amplicon sequencing

Raw sequences were analyzed using USEARCH (v10.0) (74) and QIIME v1.91 (75) as previously described (19, 32). Briefly, paired-end sequences were merged into a single sequence and the primer sequences were trimmed, and the resultant sequences were quality-filtered (maximum expected error 0.5) in USEARCH. The high-quality sequences were aligned against the *nif*H gene database to remove chimeras by UCHIME (76). OTU clustering was performed at 95% similarity using UCLUST and all singletons were removed (74). The sequences were further converted to amino acid sequences using the FunGene Pipeline of the Ribosomal Database Project, and the amino acid sequences that did not match the *nif*H protein sequence or contained termination codons were removed (56). The remaining representative sequences were annotated using the *nif*H gene database (77). This paired-end sequencing approach resulted in 17,773,861 high-quality reads, which were assembled into 18,750 OTUs. The OTU table was rarefied to 1,380 reads per sample for alpha-diversity estimates, and the cumulative sum scaling (CSS) method was for normalization before beta-diversity analyses.

### Metagenomic binning and functional analysis

To further identify N-cycling-related microbes on the phylloplane, nine maize phylloplane (based on the N treatment, three developmental stages × 3 replicates) and nine artificial leaves (three developmental stages × 3 replicates) samples from the site QJ were subjected to metagenomic sequencing using the Illumina NovaSeq platform with a paired-end protocol (15). All metagenome sequences were quality-filtered and assembled using Trimmomatic (v0.39) (78) and Megahit (v1.2.9) (79), respectively, as described previously (15). MAGs were recovered for individual metagenomic assemblies using MetaBAT2 (v2.12.1) (80) and MaxBin2 (v2.2.7) (81) according to the MetaWRAP pipeline (v1.3.1) (82), and the resulting MAGs were refined with the Bin_refinement module. The remaining MAGs were dereplicated by dRep (v2.6.2) (83), and the completeness and contamination of all MAGs were assessed using CheckM (v1.1.3) *via* the lineage-specific workflow. Based on the quality information, 58 non-redundant MAGs (includes 32 high-quality genomes) with >50% completeness and <10% contamination were retained for further analyses. Taxonomic classifications of MAGs were performed using the GTDB-Tk (v2.1.0), with reference to GTDB release R07-RS207 (including 317,542 genomes) (84, 85). MAGs were designated as potential novel species if the ANI to the closest GTDB representative genome was <0.95. The phylogenetic tree of MAGs was built by FastTree (86) using the GTDB-Tk placements and visualized using iTOL (v6.4) (87). The abundance of each MAG was calculated using Salmon (88) by the MetaWRAP pipeline. Gene annotation for MAGs was performed using Prokka (v1.14.5) (89), and the functional traits (e.g., KEGG Orthology) of MAGs were profiled by eggNOG databases (v5.0) (90). The putative secondary metabolite biosynthetic gene clusters (BGCs) from each MAG were identified using antiSMASH (v6.1.1) with the default parameters.

### Statistical analysis

A nonparametric statistical test (Kruskal–Wallis test or Wilcoxon test) was used to evaluate the alpha-diversity and the taxonomical difference of the diazotrophic community observed under different host and environmental factors. The beta-diversity was assessed by computing weighted UniFrac distance matrices and visualized using non-metric multi-dimensional (NMDS) ordinations. The relative contribution of host and environmental factors on community dissimilarity was tested with PERMANOVA or nested PERMANOVA using the *adonis* function of the “vegan” R package (91) based on weighted UniFrac distances. Source-tracking analyses were carried out to determine the potential sources of crop diazotrophs using SourceTracker (v.1.0) (92), following the methodology described in previous studies (15, 19). The phylogenetic tree of dominant taxa was built by FastTree and visualized in iTOL (87). Differential abundance analysis was performed using EdgeR’s generalized linear model (GLM) approach (93). This analysis reveals which diazotrophic taxa were significantly enriched in different compartments or under various treatments. Mantel test was performed to explore Spearman’s correlations between diazotrophic communities, soil physicochemical characteristics, and soil enzyme activities using the “vegan” package (20). The network analysis was performed using CoNet (94) in Cytoscape v3.5 (95), and only robust (Spearman’s *r* > 0.6 or *r* < –0.6) and statistically significant (*P* < 0.01) correlations were retained for visualization in Gephi (96). Random forest analyses were performed to identify the relative importance of different sub-communities in predicting crop yield using the “randomForest” R package (15, 97). All statistical analyses were carried out in R (http://www.r-project.org). More information on the PERMANOVA, network analysis, and random forest modeling analysis is detailed in our previous publication (15, 19).

## Data Availability

The raw sequencing data have been submitted to the Sequence Read Archive (SRA) under the accession number PRJNA784384 (*nif*H) and PRJNA679917 (metagenomics). The raw data and scripts associated with this study are available at Figshare (https://figshare.com/s/24f2a51568915f1138d6).

## SUPPLEMENTAL MATERIAL

Method S1 msystems.01055-23-s0001.docx Detailed description of field trial and molecular methods.

Supplemental figures msystems.01055-23-s0002.docx Fig. S1 to S7.

Table S1 msystems.01055-23-s0003.docx Effects of host (compartment, crop species, and developmental stage) and environmental (site and fertilization practice) factors on the diazotrophic community.

Table S2 msystems.01055-23-s0004.docx Spearman's correlations between diazotrophic communities in soil and rhizoplane, soil physicochemical characteristics, and soil nitrogenase activity based on Mantel tests.

ASM does not own the copyrights to Supplemental Material that may be linked to, or accessed through, an article. The authors have granted ASM a non-exclusive, world-wide license to publish the Supplemental Material files. Please contact the corresponding author directly for reuse.
