# Supplementary material for: Microbial species pool-mediated diazotrophic community assembly in crop microbiomes during plant development
Source: mSystems. 2024 Mar 19;9(4):e01055-23. doi: 10.1128/msystems.01055-23 (PMC11019923; doi:10.1128/msystems.01055-23)
Supplement: Method S1 — Detailed description of field trial and molecular methods. [file msystems.01055-23-s0001.docx]

**Supplementary methods**

**Method S1 Detailed description of field trial and molecular methods**

The two sites with contrasting climate and edaphic features are about 1800 km apart, and the field trials were managed following local regimes (maize-wheat rotation at the site XC and maize-barley rotation at the site QJ). For all treatments, 60% of N fertilizer was used as basal fertilizer and the remaining was applied before jointing stage, and both P and K fertilizers were applied as basal fertilizers. The straw applied in 80%NS (20% N reduction plus straw addition at a rate of 3000 kg ha^-1^) and 80%NSB (80%NS treatment plus biochar addition at a rate of 30,000 kg ha^-1^) treatments were from last harvest. Straws (2-3 cm lengths) were spread evenly on the surface of the soil after sowing. Biochar was pyrolytically produced from maize straw that purchased from Liao Ning Golden Future Agriculture Technology Co., Ltd, and was added evenly on the surface of the soil every two years. To estimate the impact of seasonal environmental factors (e.g., air, dust, temperature, rainfall and UV) on phylloplane diazotrophic communities, artificial plants made of plastic material were planted as “background controls” in the field, and plastic leaves were sampled at the same time with soil and plant sample collection (n=24). All samples for DNA extraction were transported to the laboratory on dry ice, and stored at –80 °C until further processing.

PCR reactions were performed in a 25μl reaction containing 12.5 μl Premix Taq DNA polymerase (TaKaRa Bio Inc., Shiga, Japan), 0.5 μl each primer (10 μM), 2 μl template DNA (~5 ng μl-1), and 9.5 μl PCR-grade water. PCR amplification was carried out using the following program: 10 min initial denaturation at 95 °C, 35 cycles of 15 s at 95 °C, 30 s at 62 °C, and 30 s at 72 °C, with a final 10-min elongation at 72 °C. PCR products were purified using the QIAquick gel extraction kit (Qiagen, USA), and sequenced on the Illumina MiSeq platform with a Paired-End protocol.
