# Supplementary material for: Microbial species pool-mediated diazotrophic community assembly in crop microbiomes during plant development
Source: mSystems. 2024 Mar 19;9(4):e01055-23. doi: 10.1128/msystems.01055-23 (PMC11019923; doi:10.1128/msystems.01055-23)
Supplement: Supplemental figures — Fig. S1 to S7. [file msystems.01055-23-s0002.docx]

**Supplementary figures**


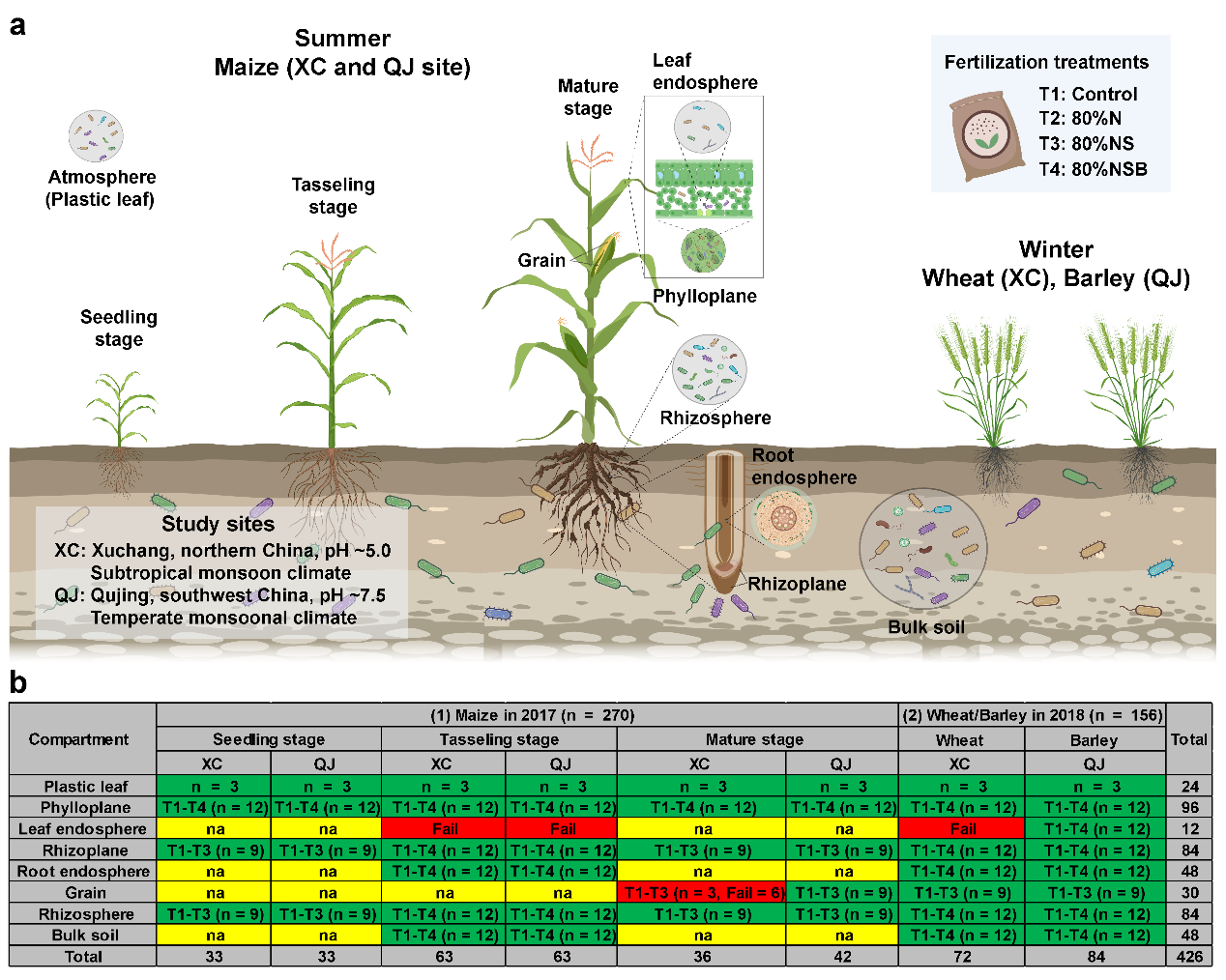


**Fig. S1** Experimental design and the layout of samples incorporating crop seasons, compartment niches, developmental stages, fertilization treatments, and sites. **(a)** Graphical illustration of the experimental design and sampling. **(b)** Samples detail for maize in 2017 **(1)** and for wheat/barley in 2018 **(2)**. “XC” represents site “Xuchang, “QJ” represents site “Qujing. “na” marked in yellow indicates that the DNA samples were not selected for *nif*H gene amplification. “Fail” marked in red represents the failure of *nif*H gene amplification. T1-T4 represent fertilization treatments: T1, Control (zero nitrogen fertilizer); T2, 80%N (20% N reduction based on local farmers’ N rate); T3, 80%NS (80%N treatment plus straw covering); T4, 80%NSB (80%NS treatment plus biochar addition).


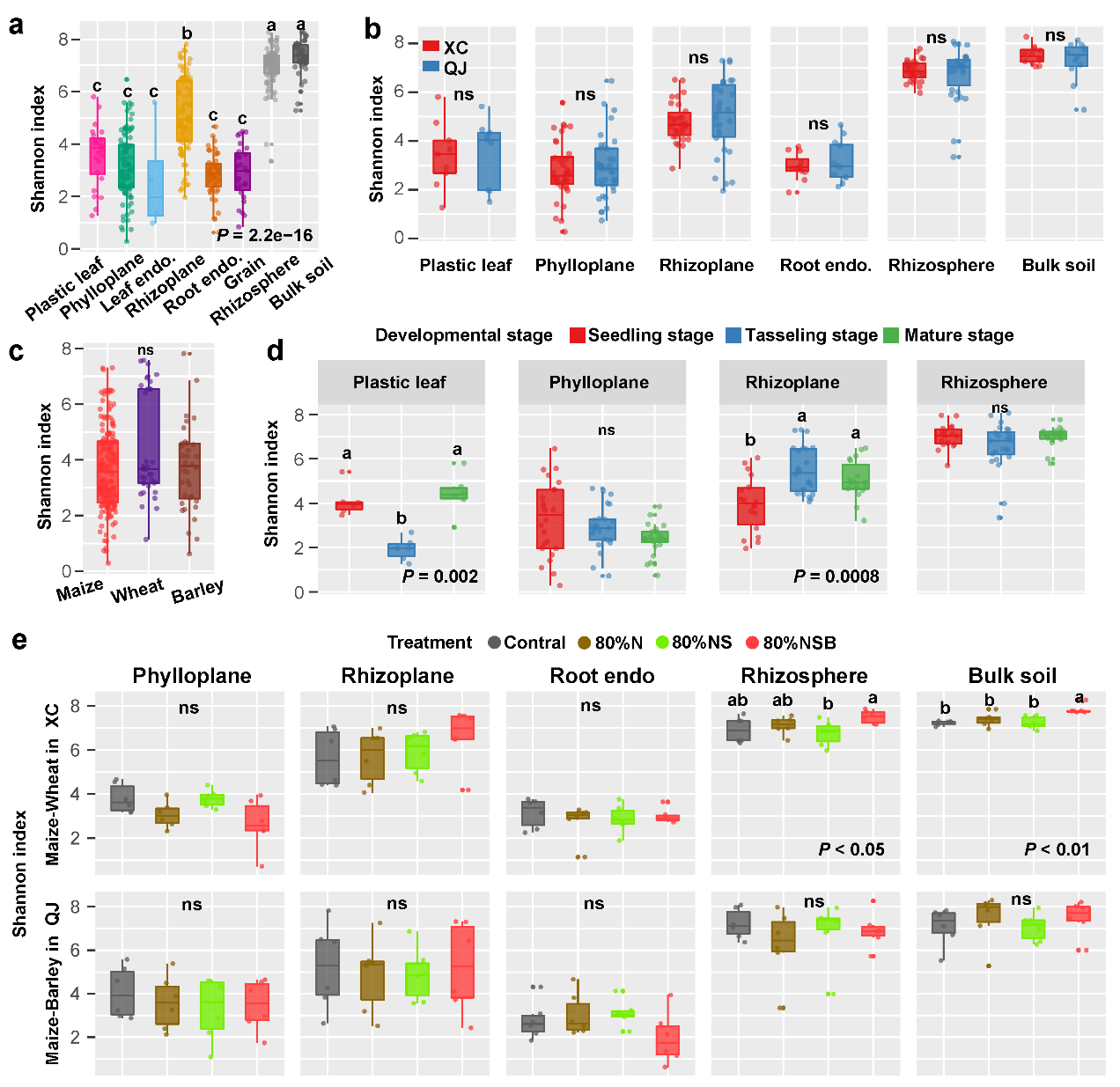


**Fig. S2** Host and environmental factors influence diazotrophic alpha diversity. Diazotrophic Shannon index in **(a)** different compartment niches, **(b)** two sites, **(c)** three crop hosts, and **(d)** three developmental stages. **(e)** Diazotrophic Shannon index in each compartment under different fertilization practices. Different letters above the boxes indicate a significant difference determined by nonparametric Kruskal Wallis test (*P* < 0.05). ns, no significance. “endo” represents the endosphere. “XC” represents site “Xuchang, Henan province”, “QJ” represents site “Qujing, Yunnan Province”.


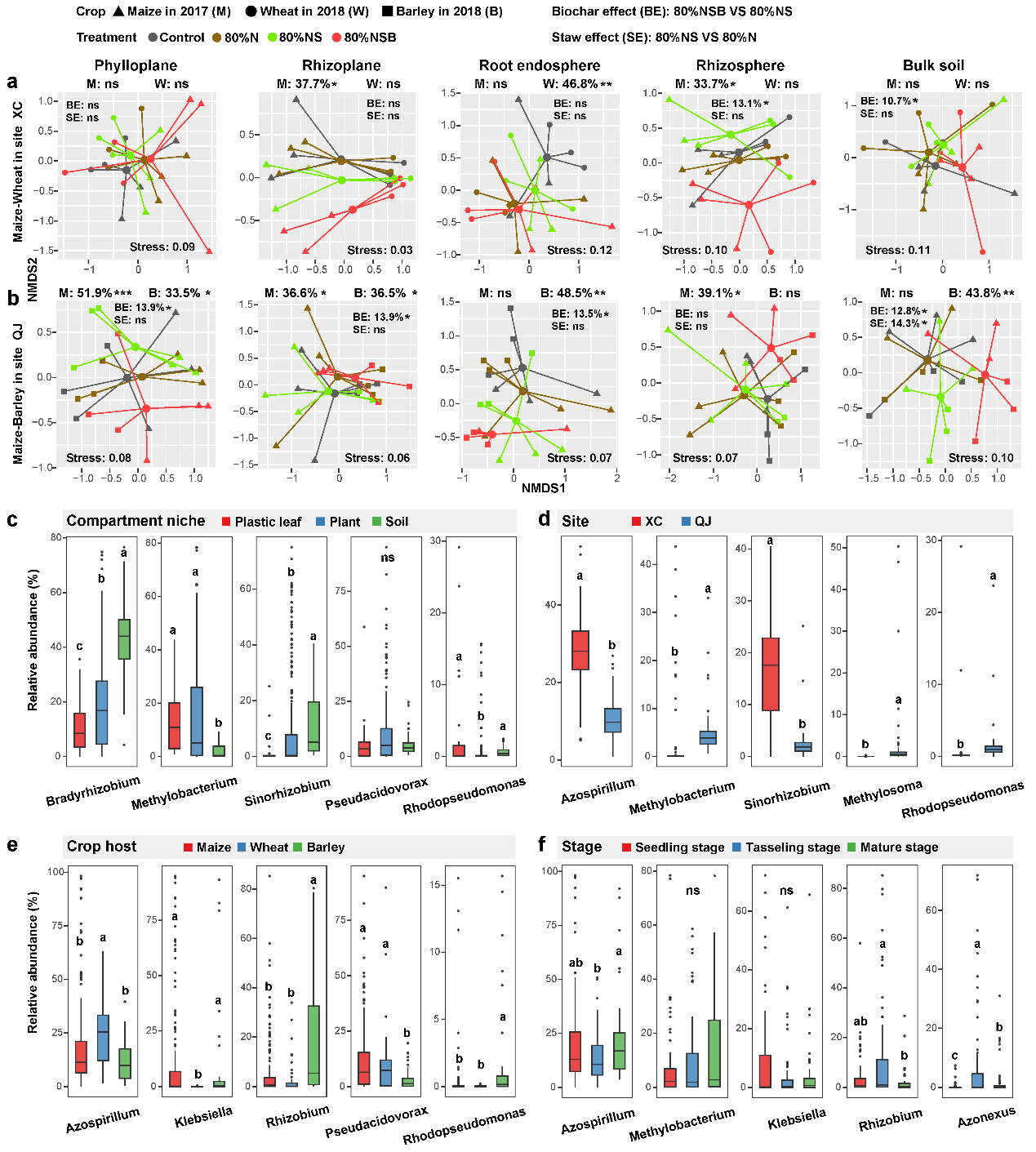


**Fig. S3** Distribution and taxonomic composition of crop-associated diazotrophic communities. NMDS ordinations showing the effects of different fertilization practices on the diazotrophic community in **(a)** maize-wheat rotation system in XC site and **(b)** maize-barley rotation system in QJ site. The relative contribution of different fertilization practices on community dissimilarity was tested with PERMANOVA (based on weighted UniFrac distances). “XC” represents site “Xuchang, Henan province”, “QJ” represents site “Qujing, Yunnan Province”. “M”, “W”, and “B” represent the variation in diazotrophic communities explained by fertilization practice in maize, wheat, and barley seasons, respectively. ns, no significance. The relative abundance of differential genera among **(c)** different compartments, **(d)** two sites, **(e)** three crops, and **(f)** three developmental stages. Through random forest analyses, we evaluated the effects of various factors (e.g., compartment and site) on the relative abundance of the top 10 diazotrophic genera and further analyzed the abundance distribution of these differential genera (top 5) across different groups. Different letters above the boxes indicate a significant difference determined by nonparametric Kruskal Wallis test (*p* < 0.05).


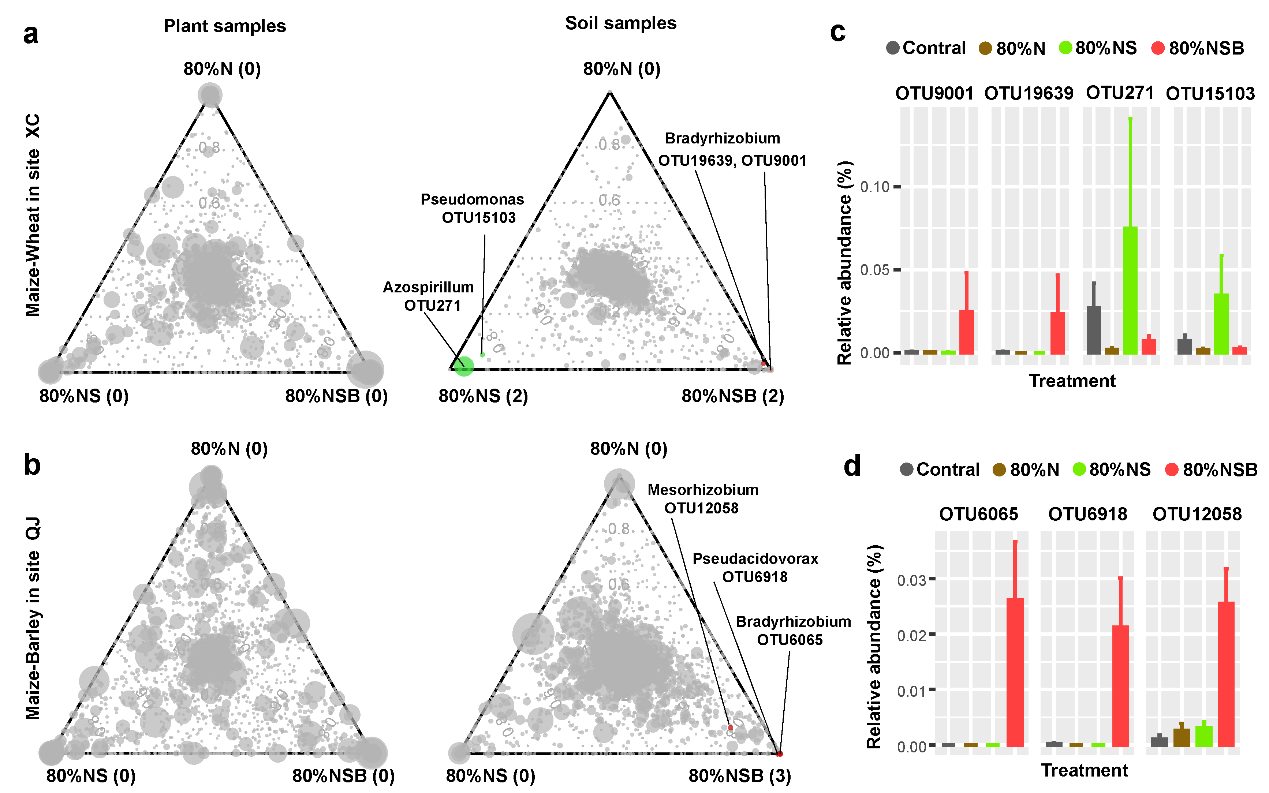


**Fig. S4** Biomarker taxa of diazotrophic communities at the OTU level under different fertilization practices. **(a**–**b)** Ternary plots depicting diazotrophic OTUs significantly enriched in different fertilization practices in plant and soil compartment niches (FDR, *P* < 0.01). Each circle represents one OTU, and the size of each circle represents its relative abundance. The green, red, and brown circles represent OTUs specifically enriched at 80%NS, 80%NSB, and 80%N treatments, respectively, whereas gray circles represent OTUs that were not significantly enriched at a specific treatment. **(c-d)** Bar plots indicate the relative abundance (mean ± SEM) of these biomarker taxa under different fertilization practices.


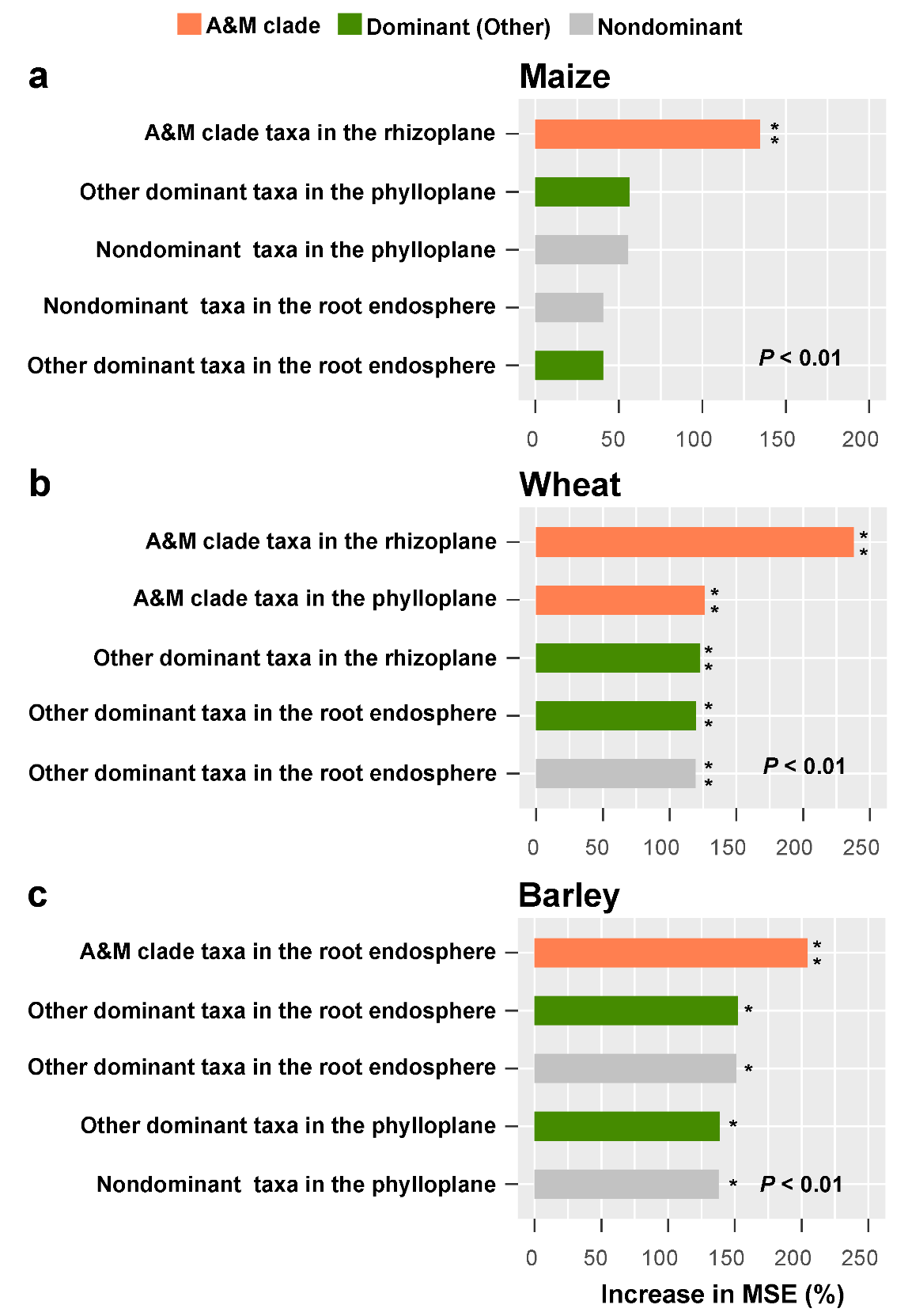


**Fig. S5** Ecological importance of diazotrophic communities in each crop. **(a-c)** Random Forest analyses identifying the relative importance of different sub-communities in predicting the crop yield. The predictors include the relative abundance of different sub-communities in leaf and root niches, and higher value of Increase in MSE represents higher importance. Increase in the percentage of MSE is equal to the increase in the mean square error. * *P* < 0.05, ** *P* < 0.01.


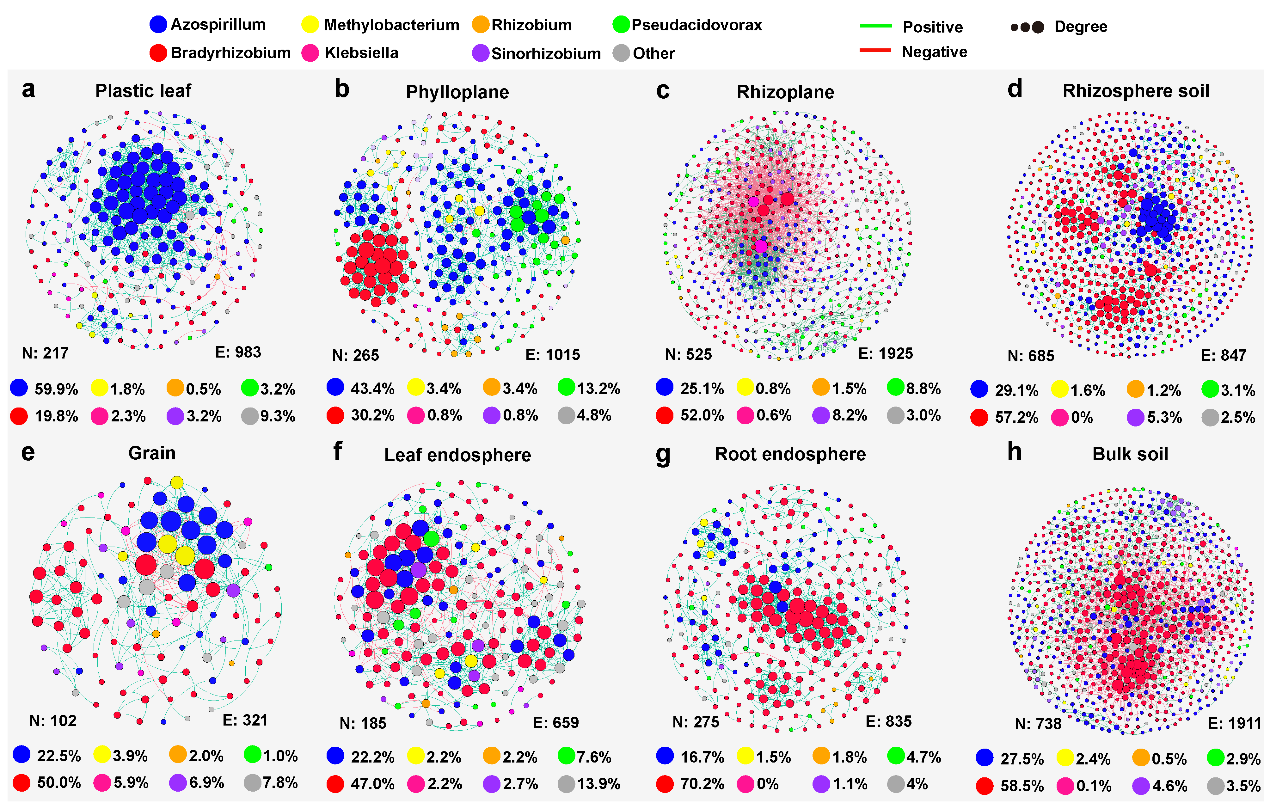


**Fig. S6** Co-occurrence networks of diazotrophic community in each compartment. **(a–h)** Co-occurrence network analysis showing microbial network patterns differ among different compartments (Plastic leaf: n = 24, phylloplane: n = 96, rhizoplane: n = 84, rhizosphere: n = 84, grain: n = 30, leaf endosphere: n = 12, root endosphere: n = 48, bulk soil: n = 48). The nodes of the network are coloured according to diazotrophic genus. The sizes of the nodes are according to the degree of connection, and the edges color represents positive (green) and negative (red) correlations. “N” represents the numbers of nodes in each network. “E” represents the numbers of edges in each network.

**
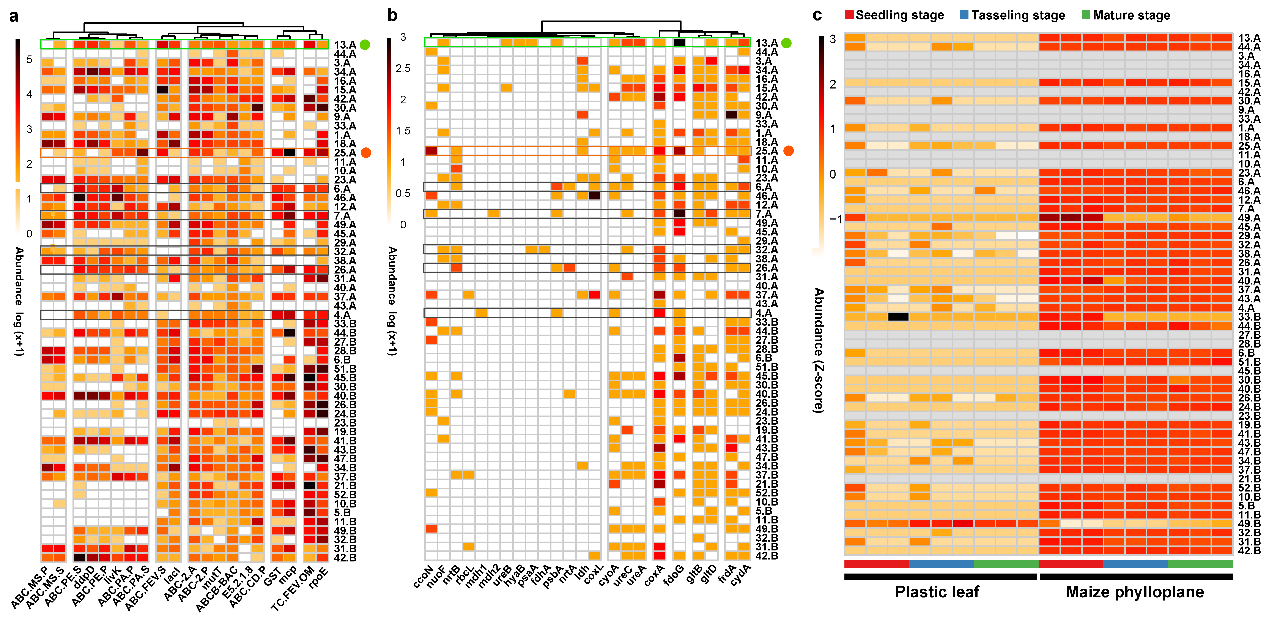
**

**Fig. S7** Metabolic characteristics and the abundance of all MAGs recovered from phylloplane microbiomes. **(a)** Heat map exhibiting the abundance of the top 20 functional genes (based on KO) from the all MAGs. **(b)** Heat map exhibiting the abundance of functional genes (based on KO) related to biogeochemical processes (e.g., carbon and nitrogen cycling) from the all MAGs. **(c)** Heat map exhibiting the abundance of all MAGs on the plastic leaf and maize phylloplane across three developmental stages.
