# Supplementary material for: Microbial species pool-mediated diazotrophic community assembly in crop microbiomes during plant development
Source: mSystems. 2024 Mar 19;9(4):e01055-23. doi: 10.1128/msystems.01055-23 (PMC11019923; doi:10.1128/msystems.01055-23)
Supplement: Table S1 — Effects of host (compartment, crop species, and developmental stage) and environmental (site and fertilization practice) factors on the diazotrophic community. [file msystems.01055-23-s0003.docx]

**Table S1** **Effects of host (compartment, crop species, and developmental stage) and environmental (site and fertilization practice) factors on the diazotrophic community**

| **Whole plant level** | | | | |
| --- | --- | --- | --- | --- |
| **Samples** | | **Variables** | **PERMANOVA** | |
|  |  |  | ***R*^2^ (%)** | ***P*** |
| All samples  (n = 426) | | Compartment niche | 21.7 | < 0.001 |
|  |  | Crop species | 2.1 | < 0.001 |
|  |  | Site | 18.8 | < 0.001 |
|  |  | Compartment/Fertilization practice | 2.6 | < 0.001 |
|  |  | Crop/Compartment | 9.4 | < 0.001 |
|  |  | Crop/Compartment/Developmental stage | 4.4 | < 0.001 |
| Crop season | Maize  (n = 270) | Compartment niche | 25.0 | < 0.001 |
|  |  | Site | 19.0 | < 0.001 |
|  |  | Compartment/Developmental stage | 6.9 | < 0.001 |
|  |  | Compartment/Fertilization practice | 2.8 | 0.670 |
|  | Wheat/  Barley  (n = 156) | Compartment niche | 29.8 | < 0.001 |
|  |  | Crop species + Site | 21.0 | < 0.001 |
|  |  | Site/Compartment | 14.7 | < 0.001 |
|  |  | Compartment/Fertilization practice | 6.7 | < 0.001 |
| Rotation system | Maize-wheat  in site XC  (n = 204) | Compartment niche | 47.3 | < 0.001 |
|  |  | Crop species | 2.2 | < 0.001 |
|  |  | Crop/Developmental stage | 2.9 | < 0.001 |
|  |  | Compartment/Developmental stage | 7.4 | < 0.001 |
|  |  | Compartment/Fertilization practice | 5.0 | 0.018 |
|  | Maize-barley  in site QJ  (n = 222) | Compartment niche | 38.1 | < 0.001 |
|  |  | Crop species | 2.9 | < 0.001 |
|  |  | Crop/Developmental stage | 2.4 | < 0.001 |
|  |  | Compartment/Developmental stage | 5.4 | < 0.001 |
|  |  | Compartment/Fertilization practice | 7.4 | < 0.001 |

| **Compartment level** | | | | |
| --- | --- | --- | --- | --- |
| **Samples** | | **Variables** | **PERMANOVA** | |
|  |  |  | ***R*^2^ (%)** | ***P*** |
| Maize samples across three developmental stages | Phylloplane  (n = 72) | Developmental stage | 16.8 | < 0.001 |
|  |  | Site | 10.8 | < 0.001 |
|  |  | Fertilization practice | 4.3 | 0.09 |
|  | Rhizoplane  (n = 60) | Developmental stage | 6.8 | 0.03 |
|  |  | Site | 50.5 | < 0.001 |
|  |  | Fertilization practice | 3.5 | 0.132 |
|  | Rhizosphere  (n = 60) | Developmental stage | 2.5 | 0.015 |
|  |  | Site | 79.1 | < 0.001 |
|  |  | Fertilization practice | 1.3 | 0.253 |
|  | Plastic leaf  (n = 18) | Developmental stage | 36.4 | < 0.001 |
|  |  | Site | 15.4 | < 0.001 |
| All samples  (Tasseling stage of maize season + Wheat/Barley season) | Phylloplane  (n = 48) | Site/Crop species | 21.2 | < 0.001 |
|  |  | Site | 17.3 | < 0.001 |
|  |  | Fertilization practice | 7.2 | 0.009 |
|  | Rhizoplane  (n = 48) | Site/Crop species | 14.9 | < 0.001 |
|  |  | Site | 62.7 | < 0.001 |
|  |  | Fertilization practice | 2.9 | 0.003 |
|  | Root endosphere  (n = 48) | Site/Crop species | 8.1 | 0.003 |
|  |  | Site | 10.9 | 0.009 |
|  |  | Fertilization practice | 5.3 | 0.57 |
|  | Rhizosphere soil  (n = 48) | Site/Crop species | 6.8 | < 0.001 |
|  |  | Site | 77.9 | < 0.001 |
|  |  | Fertilization practice | 1.4 | 0.07 |
|  | Bulk soil  (n = 48) | Site/Crop species | 1.7 | < 0.001 |
|  |  | Site | 85.4 | < 0.001 |
|  |  | Fertilization practice | 1.3 | < 0.001 |
|  | Plastic leaf  (n = 12) | Crop season | 40.3 | 0.008 |
|  |  | Site | 12.8 | 0.008 |
| All samples | Plastic leaf  (n = 24) | Site | 16.9 | < 0.001 |

| **Comparison of diazotrophic community structure between different groups** | | | |
| --- | --- | --- | --- |
| **Variables** | **Comparison** | **PERMANOVA** | |
|  |  | ***R*^2^ (%)** | ***P*** |
| Compartment  (All samples) | Phylloplane VS Plastic leaf (n = 120) | 2.2 | 0.011 |
|  | Phylloplane VS Leaf endosphere (n = 108) | 8.3 | < 0.001 |
|  | Phylloplane VS Grain (n = 126) | 3.2 | < 0.001 |
|  | Phylloplane VS Rhizoplane (n = 180) | 18.8 | < 0.001 |
|  | Phylloplane VS Root endosphere (n = 144) | 8.2 | < 0.001 |
|  | Phylloplane VS Rhizosphere (n = 180) | 22.4 | < 0.001 |
|  | Phylloplane VS Bulk soil (n = 144) | 21.4 | < 0.001 |
|  | Plastic leaf VS Leaf endosphere (n = 36) | 17.0 | < 0.001 |
|  | Plastic leaf VS Grain (n = 54) | 5.0 | 0.007 |
|  | Plastic leaf VS Rhizoplane (n = 108) | 15.9 | < 0.001 |
|  | Plastic leaf VS Root endosphere (n = 72) | 13.0 | < 0.001 |
|  | Plastic leaf VS Rhizosphere (n = 108) | 18.4 | < 0.001 |
|  | Plastic leaf VS Bulk soil (n = 72) | 22.8 | < 0.001 |
|  | Leaf endosphere VS Grain (n = 42) | 12.4 | < 0.001 |
|  | Leaf endosphere VS Rhizoplane (n = 96) | 6.2 | 0.03 |
|  | Leaf endosphere VS Root endosphere (n = 60) | 15.6 | < 0.001 |
|  | Leaf endosphere VS Rhizosphere (n = 96) | 6.6 | 0.006 |
|  | Leaf endosphere VS Bulk soil (n = 60) | 10.1 | 0.003 |
|  | Grain VS Rhizoplane (n = 114) | 14.8 | < 0.001 |
|  | Grain VS Root endosphere (n = 78) | 10.7 | < 0.001 |
|  | Grain VS Rhizosphere (n = 114) | 15.9 | < 0.001 |
|  | Grain VS Bulk soil (n = 78) | 18.8 | < 0.001 |
|  | Rhizoplane VS Root endosphere (n = 132) | 13.9 | < 0.001 |
|  | Rhizoplane VS Rhizosphere (n = 168) | 4.8 | < 0.001 |
|  | Rhizoplane VS Bulk soil (n = 132) | 5.9 | < 0.001 |
|  | Root endosphere VS Rhizosphere (n = 132) | 19.5 | < 0.001 |
|  | Root endosphere VS Bulk soil (n = 96) | 22.0 | < 0.001 |
|  | Rhizosphere VS Bulk soil (n = 132) | 1.0 | 0.217 |
| Crop  (Phylloplane, Rhizoplane, Root endosphere) | Maize VS Wheat (n = 192) | 3.8 | < 0.001 |
|  | Maize VS Barley (n = 192) | 5.6 | < 0.001 |
|  | Wheat VS Barley (including site effect, n = 72) | 17.7 | < 0.001 |
| Crop  (Phylloplane) | Maize (XC) VS Wheat (XC) (n = 24) | 20.5 | < 0.001 |
|  | Maize (QJ) VS Barley (QJ) (n = 24) | 30.5 | < 0.001 |
|  | Maize (XC) VS Maize (QJ) (n = 24) | 20.6 | < 0.001 |
| Crop  (Rhizoplane) | Maize (XC) VS Wheat (XC) (n = 24) | 58.8 | < 0.001 |
|  | Maize (QJ) VS Barley (QJ) (n = 24) | 32.9 | < 0.001 |
|  | Maize (XC) VS Maize (QJ) (n = 24) | 65.1 | < 0.001 |
| Crop  (Root endosphere) | Maize (XC) VS Wheat (XC) (n = 24) | 6.6 | 0.09 |
|  | Maize (QJ) VS Barley (QJ) (n = 24) | 11.8 | 0.003 |
|  | Maize (XC) VS Maize (QJ) (n = 24) | 15.5 | < 0.001 |
| Crop  (Rhizosphere) | Maize (XC) VS Wheat (XC) (n = 24) | 29.3 | < 0.001 |
|  | Maize (QJ) VS Barley (QJ) (n = 24) | 31.1 | < 0.001 |
|  | Maize (XC) VS Maize (QJ) (n = 24) | 79.7 | < 0.001 |
| Crop  (Bulk soil) | Maize (XC) VS Wheat (XC) (n = 24) | 8.7 | 0.019 |
|  | Maize (QJ) VS Barley (QJ) (n = 24) | 12.3 | < 0.001 |
|  | Maize (XC) VS Maize (QJ) (n = 24) | 87.0 | < 0.001 |
| Developmental stage  (Phylloplane) | Seedling stage VS Tasseling stage (n = 48) | 12.3 | < 0.001 |
|  | Seedling stage VS Mature stage (n = 48) | 20.2 | < 0.001 |
|  | Tasseling stage VS Mature stage (n = 48) | 5.7 | 0.003 |
| Developmental stage  (Rhizoplane) | Seedling stage VS Tasseling stage (n = 42) | 5.1 | 0.118 |
|  | Seedling stage VS Mature stage (n = 36) | 6.5 | 0.086 |
|  | Tasseling stage VS Mature stage (n = 42) | 4.3 | 0.126 |
| Developmental stage  (Rhizosphere) | Seedling stage VS Tasseling stage (n = 42) | 2.7 | 0.391 |
|  | Seedling stage VS Mature stage (n = 36) | 2.1 | 0.326 |
|  | Tasseling stage VS Mature stage (n = 42) | 1.1 | 0.683 |

The relative contribution of host and environmental factors on the diazotrophic community was tested with PERMANOVA or nested PERMANOVA (based on weighted UniFrac distance).
