# Supplementary material for: Microbial species pool-mediated diazotrophic community assembly in crop microbiomes during plant development
Source: mSystems. 2024 Mar 19;9(4):e01055-23. doi: 10.1128/msystems.01055-23 (PMC11019923; doi:10.1128/msystems.01055-23)
Supplement: Table S2 — Spearman's correlations between diazotrophic communities in soil and rhizoplane, soil physicochemical characteristics, and soil nitrogenase activity based on Mantel tests. [file msystems.01055-23-s0004.docx]

**Table S2 Spearman’s correlations between diazotrophic communities in soil and rhizoplane, soil physicochemical characteristics, and soil nitrogenase activity based on Mantel tests**

| Variables | | Rhizoplane | | | | | |  | Rhizosphere | | | | | |
| --- | --- | --- | --- | --- | --- | --- | --- | --- | --- | --- | --- | --- | --- | --- |
|  |  | All | | Dominant | | Non-dominant | |  | All | | Dominant | | Non-dominant | |
|  |  | *r* | *P* | *r* | *P* | *r* | *P* |  | *r* | *P* | *r* | *P* | *r* | *P* |
| Environmental  factors | **pH** | 0.83 | < 0.001 | 0.82 | < 0.001 | 0.82 | < 0.001 |  | 0.83 | < 0.001 | 0.85 | < 0.001 | 0.82 | < 0.001 |
|  | NH_4_^+^-N | 0.06 | 0.10 | 0.04 | 0.17 | 0.08 | 0.06 |  | 0.001 | 0.48 | 0.06 | 0.14 | 0.01 | 0.58 |
|  | **NO_3_^-^-N** | 0.33 | < 0.001 | 0.32 | < 0.001 | 0.32 | < 0.001 |  | 0.28 | < 0.001 | 0.35 | < 0.001 | 0.25 | < 0.001 |
|  | **DOC** | 0.43 | < 0.001 | 0.43 | < 0.001 | 0.43 | 0.01 |  | 0.40 | < 0.001 | 0.43 | < 0.001 | 0.40 | 0.01 |
|  | DON | 0.27 | < 0.001 | 0.27 | < 0.001 | 0.27 | 0.002 |  | 0.26 | 0.002 | 0.30 | < 0.001 | 0.26 | 0.002 |
|  | **NA** | **0.12** | **0.003** | **0.15** | **0.003** | **0.10** | **0.008** |  | **0.13** | **0.003** | **0.14** | **0.002** | **0.13** | **0.003** |

All, whole diazotrophic community; Dominant, dominant diazotrophic sub-community for maize; No-dominant, non-dominant diazotrophic sub-community. The significances are tested based on 999 permutations. DOC, Dissolved organic carbon. DON, Dissolved organic nitrogen. NA, Nitrogenase activity.
